# Arabidopsis UMAMIT24 and 25 are amino acid exporters involved in seed loading

**DOI:** 10.1093/jxb/ery302

**Published:** 2018-08-24

**Authors:** Julien Besnard, Chengsong Zhao, Jean-Christophe Avice, Stanislav Vitha, Ayumi Hyodo, Guillaume Pilot, Sakiko Okumoto

**Affiliations:** 1 Department of Soil and Crop, Texas A&M, College Station, TX, USA; 2 School of Plant and Environmental Sciences, Virginia Tech, Blacksburg, VA, USA; 3 UMR INRA - UCBN 950 EVA, UFR des Sciences, Département de Biologie, Université de Caen Normandie, Esplanade de la Paix, Caen cedex, France; 4 Microscopy and Imaging Center, Texas A&M, College Station, TX, USA; 5 Stable Isotopes for Biosphere Science Laboratory, Texas A&M, College Station, TX, USA

**Keywords:** Amino acids, Arabidopsis, membrane export, nitrogen, seed, transporter, UMAMIT

## Abstract

Phloem-derived amino acids are the major source of nitrogen supplied to developing seeds. Amino acid transfer from the maternal to the filial tissue requires at least one cellular export step from the maternal tissue prior to the import into the symplasmically isolated embryo. Some members of UMAMIT (usually multiple acids move in an out transporter) family (UMAMIT11, 14, 18, 28, and 29) have previously been implicated in this process. Here we show that additional members of the UMAMIT family, UMAMIT24 and UMAMIT25, also function in amino acid transfer in developing seeds. Using a recently published yeast-based assay allowing detection of amino acid secretion, we showed that UMAMIT24 and UMAMIT25 promote export of a broad range of amino acids in yeast. In plants, UMAMIT24 and UMAMIT25 are expressed in distinct tissues within developing seeds; UMAMIT24 is mainly expressed in the chalazal seed coat and localized on the tonoplast, whereas the plasma membrane-localized UMAMIT25 is expressed in endosperm cells. Seed amino acid contents of *umamit24* and *umamit25* knockout lines were both decreased during embryogenesis compared with the wild type, but recovered in the mature seeds without any deleterious effect on yield. The results suggest that UMAMIT24 and 25 play different roles in amino acid translocation from the maternal to filial tissue; UMAMIT24 could have a role in temporary storage of amino acids in the chalaza, while UMAMIT25 would mediate amino acid export from the endosperm, the last step before amino acids are taken up by the developing embryo.

## Teaser

UMAMIT24 and UMAMIT25 are expressed in distinct seed tissue during Arabidopsis embryogenesis, and are both involved in amino acid transfer to the seed.

## Introduction

Transfer of nutrients from the mother plant to the reproductive tissues is critical to ensure proper seed development. In most plant species, phloem-derived amino acids are the main form of nitrogenous compounds transported to the seeds and are stored as proteins, necessary for seed metabolism during seed maturation and germination (Pate *et al.*, 1975; Atkins *et al.*, 1979). The quantity of amino acids delivered through the phloem to the developing seed correlates with seed protein and lipid content, suggesting that phloem amino acid concentration is an important factor in determining seed quality traits such as protein content (Saravitz and Raper, 1995; Lohaus and Moellers, 2000). Manipulation of phloem amino acid content through loss-of-function mutations and enhanced gene expression of amino acid transporters also affects seed protein content and yield, supporting the importance of phloem-derived amino acids in determination of yield (Zhang *et al.*, 2010; Santiago and Tegeder, 2016, 2017).

Once delivered to the reproductive tissues through the phloem, amino acids are released from the mother tissue before they are taken up by the symplasmically isolated daughter tissues via H^+^/amino acid symporters (Schmidt *et al.*, 2007; Tan *et al.*, 2008; Sanders *et al.*, 2009; Tegeder, 2014; Zhang *et al.*, 2015; reviewed in Karmann *et al.*, 2018). Studies using the ‘empty seed coat’ technique in legumes revealed that amino acids are released from the seed coat by a proton gradient-independent and transporter-mediated mechanism (De Jong *et al.*, 1997). For a long time, the molecular mechanism for this amino acid transport remained unknown (Okumoto and Pilot, 2011). However, several members of the plant-specific UMAMIT (usually multiple acid move in and out transporter) family were shown to mediate proton gradient-insensitive amino acid transport and represent a potential mechanism for release of amino acids from the mother tissues to the developing seeds. Indeed, several UMAMIT proteins have been suggested to play a role in the seed loading process: UMAMIT18 is expressed in the chalazal seed coat within Arabidopsis seeds during the early stage of embryogenesis, and the loss-of-function mutant accumulates less amino acids in the developing siliques (Ladwig *et al.*, 2012). Likewise, UMAMIT11, UMAMIT14, UMAMIT28, and UMAMIT29 are expressed within developing seeds and are involved in amino acid loading in siliques during early stages of embryogenesis (Müller *et al.*, 2015). For each of the UMAMIT proteins found so far to be expressed within the seed, the phenotype caused by a single loss-of-function mutation is relatively benign (i.e. no loss of seed viability), suggesting the involvement of multiple amino acid exporters, including previously uncharacterized UMAMITs, in amino acid transport to the embryo.

In this study, we report the characterization of the role of two additional members of the UMAMIT family in Arabidopsis, UMAMIT24 and UMAMIT25, in the seed filling process. The expression patterns of UMAMIT24 and UMAMIT25 differ during the seed filling stage, suggesting that they play distinct roles in seed filling of amino acids during embryogenesis.

## Materials and methods

### Plant culture

Arabidopsis (Col-0) plants were grown in long days [16 h light at 125 µmol m^–2^ s^–1^, 60% humidity, 23/18 °C day/night in a 2:1 (v:v) mix of SUN GRO SUNSHINE LC1 GROWER MIX^®^ and vermiculite]. Plants were watered with 0.15 g l^–1^ MiracleGro^®^ fertilizer (24:8:16, N:P:K) three times a week. For kanamycin selection, seeds were sown on MS medium (half-strength Murashige and Skoog medium supplemented with 30 mM sucrose and 0.8% agarose with pH adjusted to 5.8 with KOH) containing 50 µg ml^–1^ kanamycin. Hygromycin selection was performed as described by Harrison *et al.* (2006) using MS medium complemented with 20 µg ml^–1^ hygromycin. Kanamycin- and hygromycin-resistant plants were transferred to the long-day condition described above after 1 week of selection. Plants were transformed using the floral dip method with *Agrobacterium tumefaciens* strain GV3101 (pMP90) (Clough and Bent, 1998). The *umamit24* T-DNA line GABI_012H03 (*umamit24-1*) was obtained from the GABI-Kat III collection (Kleinboelting *et al.*, 2012). The *umamit25* T-DNA line SALK_140423 (*umamit25-1*) was obtained from the Arabidopsis Biological Resource Center (Alonso *et al.*, 2003). Both T-DNA insertions were confirmed by PCR (see Supplementary Fig. S1 at *JXB* online) and quantitative PCR (Supplementary Table S1). To generate the complemented lines, *umamit24-1* and *umamit25-1* were transformed with *UMAMIT24* promoter–*UMAMIT24* genomic DNA (gDNA)–green fluorescent protein (GFP) or *UMAMIT25* promoter–*UMAMIT25* gDNA–GFP, respectively. To generate the UMAMIT24 and UMAMIT25 lines used for the analysis of spatiotemporal expression of UMAMIT24–GFP and UMAMIT–25GFP as well the subcellular localization of UMAMIT24–GFP, wild-type (WT) plants were transformed with *UMAMIT24* promoter–*UMAMIT24* gDNA–GFP or *UMAMIT25* promoter–*UMAMIT25* gDNA–GFP. To generate the overexpressor lines used for subcellular localization of UMAMIT25–GFP, WT plants were transformed with 35S:*UMAMIT25*-GFP.

### DNA constructs

The *UMAMIT24* promoter (2546 bp upstream from ATG) and gDNA or *UMAMIT24* gDNA were amplified by PCR from Col-0 gDNA. The PCR fragments were cloned into pDONRZeo (Life Technologies, USA). The *UMAMIT24* promoter–*UMAMIT24* gDNA or *UMAMIT24* gDNA was recombined into the destination vectors pWGkan2 or pPWGYTkan, respectively, derivatives of pJHA212K (Yoo *et al.*, 2005), leading to N-terminal translational fusions with GFP or a GFP–Myc tag protein, placed under the control of the *UMAMIT24* or the *Cauliflower mosic virus* (CaMV) 35S promoter, respectively. pWGKan2 carrying *UMAMIT24* promoter–*UMAMIT24* gDNA was used to generate the lines used to visualize spatiotemporal expression and subcellular localization of UMAMIT24–GFP, as well as the *umamit24-1/*UMAMIT24 complemented line. The same steps were used for the cloning of UMAMIT25 using *UMAMIT25* promoter (2963 bp upstream from ATG)–*UMAMIT25* gDNA or *UMAMIT25* gDNA, as well as for the generation of *umamit25-1/*UMAMIT25 complemented line. For yeast uptake studies, *UMAMIT23* cDNA, *UMAMIT24* cDNA, and *UMAMIT25* cDNA were cloned into the pDONRZeo vector and transferred to the yeast expression vector pDR196-Ws (Besnard *et al.*, 2016; Loqué *et al.*, 2007). All entry clones were sequenced prior to use. Sequence and map information for all constructs are available upon request. Primers used for cloning and quantitative reverse transcription–PCR (qRT–PCR) are listed in Supplementary Table S2.

### Analytical methods

Acquisition and harvesting of siliques at 7, 10, and 14 d after pollination (DAP) were performed as described in Supplementary Fig. S2. Free amino acids were extracted by adding 200 μl of chloroform and 10 mM HCl (1:1 mixture) to 1–2 mg of lyophilized plant tissue, and the aqueous phase was collected. Samples were derivatized using *o*-phthalaldehyde (OPA; Agilent^®^ #5061-3335) and 9-fluoromethyl-chloroformate (FMOC; Agilent^®^ #5061-3337), and the derivatized amino acids were separated by reverse phase HPLC using an Agilent 1260 liquid chromatograph. Amino acids were detected by fluorescence using an in-line fluorescence detector (G1321B). Seed and pericarp (defined here as the silique minus the seeds) protein extraction and quantification was performed as described in Gallardo *et al.* (2002) using 2 mg of lyophilized tissue. The nitrogen content of seed protein was deduced using the average nitrogen content of nine proteins belonging to the two major families of seed storage proteins (napins and cruciferins) in Arabidopsis (17.7%, w/w). Pericarp nitrogen content was calculated using a commonly used conversion factor for plant tissues (16%, w/w) (Jones, 1941).

### Glutamine and sucrose transfer assay in isolated siliques

Three siliques were excised from the plant at 10 DAP (Supplementary Fig. S2), aligned, and cut using a razor blade so that the pedicels all have a length of 5 mm. The three siliques were transferred into a 0.25 ml PCR tube with their pedicel in contact with 5 µl of a solution containing 20 mM sucrose and 2 mM Gln. At the beginning of the experiment, the solution was replaced with 5 µl of 20 mM sucrose and 2 mM Gln with 5% and 10% isotopic excess of U^13^C sucrose and U^15^N Gln, respectively (or no isotopic excess for the negative controls), and the PCR tube was closed. Four hours later, the solution was removed from the PCR tube, the pedicel was excised from each silique, and the three siliques were lyophilized. The seeds were separated from the pericarp tissue, and isotopic enrichment and percentage of C and N (δ^13^C, δ^15^N, %N, and %C, respectively) in seeds were determined in the Stable Isotopes for Biosphere Science (SIBS) Laboratory, Texas A&M University, using a Costech^®^ Elemental Combustion System coupled to a Thermo Scientific^®^ Delta V Advantage stable isotope mass spectrometer and Conflo IV^®^ in continuous flow (helium) mode. Nitrogen and carbon contents (%) were calculated in w/w of tissue. Isotopic enrichment was determined by subtracting the ^13^C and ^15^N content of each line’s negative control from the ^13^C and ^15^N of the samples.

### Yeast secretion assay

Determination of the amount of amino acids secreted in the medium by yeast cells 22Δ10α (genotype MATα gap1-1 put4-1 uga4-1 can1::HisG lyp1-alp1::HisG hip1::HisG dip5::HisG gnp1Δ agp1Δ ura3-1) expressing the UMAMIT proteins was performed as described in Besnard *et al.* (2016). Briefly, cells were grown for 22 h in a minimal medium (Jacobs *et al.*, 1980) supplemented with 2.5 mM (NH_4_)_2_SO_4_, the OD was recorded, the medium was separated from the cells by filtration, and then clarified on a 10 kDa exclusion membrane. The amino acid content of the medium was determined by UPLC as described in Besnard *et al.* (2016).

### RNA extraction and qRT–PCR

Total RNAs were extracted according to Downing *et al.* (1992) using ~25 mg of fresh seeds separated from pericarp tissue. Samples of 2 µg of total RNAs were used for cDNA synthesis with random primers using a High Capacity cDNA Reverse Transcription Kit (Applied Biosystems^®^, USA). qRT–PCR was performed using SsoAdvanced Universal Inhibitor-Tolerant SYBR Green Supermix in a CFX96 Touch Real-Time PCR machine (Biorad^®^) according to manufacturer’s recommendations.

### Transient expression in Arabidopsis

Proteins were transiently expressed in Arabidopsis according to Wu *et al.* (2014) using *Agrobacterium* strain C58C1 (pCH32) transformed with pPWGYTkan containing the *UMAMIT24* genomic sequence without the stop codon, and the binary vector ER-RB CD3-960 containing the HDEL–mCherry coding sequence for endoplasmic reticulum (ER) visualization (Nelson *et al.*, 2007).

### Seed protoplasting

At 14 DAP, WT or *UMAMIT24* promoter–*UMAMIT24* gDNA–GFP seeds were isolated from the pericarp tissue, and digested according to Weigel and Glazebrook (2002) using 10 mg ml^–1^ cellulase, 5 mg ml^–1^ macerozyme, and 10 mg ml^–1^ driselase for 6 h. Protoplasts of chalazal seed coat detached from the seed were then imaged using the microscopy setting described below.

### Seed fixation

Siliques at 14 DAP were fixed for 4 h in 3% (w/v) formaldehyde, then microwaved at 250 W in a microwave processor (Pelco Biowave, Ted Pella, USA) using a 2–2–2 program (2 min on, 2 min off, 2 min on) under vacuum. The samples were kept in an ice bath during the microwave treatment. The siliques were then washed first in water then in phosphate-bufferedd saline (PBS; pH. 8.0). The seeds were extracted, microwaved on the same setting as above for 1 min, then incubated in 70% 2,2'-thiodiethanol (Sigma^®^, #88559) under gentle shaking overnight to clear the tissue, as described by Musielak *et al.* (2016) Seeds were stored at 4 °C until imaging.

### Fluorescence and confocal microscopy imaging

Images and *Z*-stacks of UMAMIT24–GFP and UMAMIT25–GFP for localization of UMAMIT24–GFP and UMAMIT25–GFP within the seed were taken using a Zeiss^®^ LSM 880 confocal laser-scanning microscope. Spatiotemporal expression of UMAMIT24–GFP and UMAMIT25–GFP was examined using an Olympus^®^ SZXZRFL3 stereomicroscope attached with the Olympus DP71 digital camera (Olympus^®^ America, NY, USA). Subcellular localization of UMAMIT24–GFP or UMAMIT25–GFP was imaged using an Olympus^®^ FV1000 confocal microscope equipped with an UPLSAPO ×60/1.2 water immersion objective (for protoplats) or a ×20/0.85 oil immersion objective (for thiodiethanol-cleared tissue), with the confocal pinhole size corresponding to 1 Airy unit. All images were taken using wavelengths as follows: GFP (excitation 488 nm, emission 500–530 nm), mCherry (excitation 543 nm, emission 565–615 nm), chlorophyll (excitation 488 nm, emission >650 nm), or Syto82 (excitation 543 nm, emission 565–615 nm), and merged using ZEN^®^ software (Zeiss), Olympus^®^ Fluoview (Olympus^®^), and ImageJ.

### Statistical analyses

*T*-tests were performed in Microsoft^®^ Excel. One-way ANOVA in conjunction with Tukey’s test were used to determine significant differences between samples in JMP^®^ (SAS, USA). Principal components analyses (PCAs) were performed using the Stats package in R (Venables and Ripley, 2002).

### Accession numbers

Accession numbers for UMAMIT24 (AT1G25270) and UMAMIT25 (AT1G09380) have been assigned by The Arabidopsis Information Resource (TAIR; http://www.arabidopsis.org/).

## Results

### UMAMIT24 and UMAMIT25 function as amino acid exporters in yeast cells

**Fig. 1.**
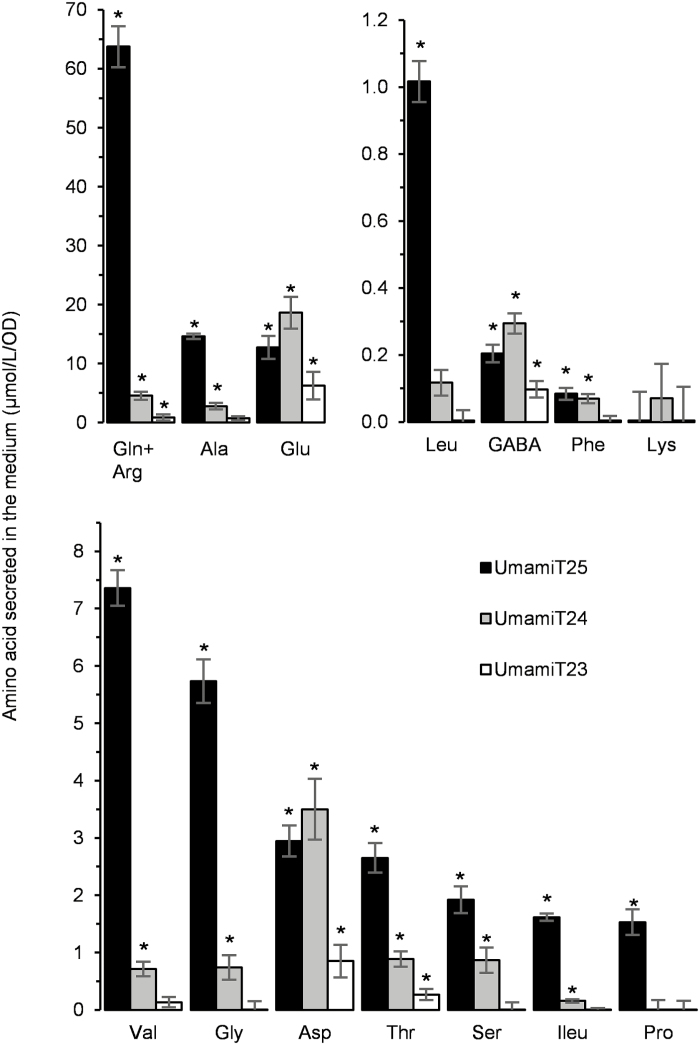
Secretion of amino acids in the medium by UMAMIT-expressing yeast cells. 22Δ10α cells were transformed with the empty vector pDR196-Ws from which the gateway cassette has been removed, or containing *UMAMIT25, UMAMIT24*, or *UMAMIT23*. Cells were grown for 22 h in liquid medium; then the amino acid composition of the medium was determined by UPLC. The Arg peak could not be resolved from the large Gln peak in samples so these amino acids are presented by a single bar. Concentrations found in the medium were divided by the OD of the culture, and the value obtained for the empty vector was subtracted. Error bars correspond to the SE (*n*=4 biological replicates). Significant differences (*P*<0.01) compared with the empty vector are indicated by an asterisk according to *t*-test.

Using a *Saccharomyces cerevisiae* strain 22∆10α, which lacks 10 of the endogenous amino acid transporters (Besnard *et al.*, 2016), we screened 44 UMAMITs from Arabidopsis that were capable of exporting Gln into the growth medium (SO, unpublished). This screen identified UMAMIT25 as a potential amino acid exporter, and phylogenetic analysis revealed two closely related paralogs, UMAMIT23 and UMAMIT24 (Denancé *et al.*, 2014). Publicly available microarray data showed that UMAMIT23, UMAMIT24, and UMAMIT25 are almost exclusively expressed in the developing siliques and seeds (Schmid *et al.*, 2005), in which amino acid exporters play an important role in nitrogen transfer to the filial tissue. In order to examine whether these three UMAMITs function as exporters, they were expressed in 22∆10α (Besnard *et al.*, 2016). The cells were grown for 22 h in a medium lacking amino acids, with ammonium as the sole nitrogen source, and the amino acid content in the medium was determined. If any of the UMAMIT genes encode an amino acid exporter, the amino acid content in the medium from the cells expressing these genes was expected to be higher than for control cells carrying the empty vector (Velasco *et al.*, 2004; Ladwig *et al.*, 2012; Besnard *et al.*, 2016). Cells expressing UMAMIT24 or UMAMIT25 showed a significantly higher secretion of most of the detectable amino acids compared with control cells (Fig. 1). UMAMIT25-expressing cells secreted more than five times as much Gln, Val, Ile, Gly, and Leu (47×, 10.5×, 9×, 5.2×, and 5×, respectively) as control cells, while UMAMIT24-expressing cells secreted amino acids to a lesser degree but significantly higher than control cells (between 5.4× for Gln and <2.5× for the other amino acids relative to the control cells). The two transporters thus seem to have different substrate specificities; the amino acids mostly secreted by the UMAMIT25- and UMAMIT24-expressing yeasts are, respectively, Gln+Arg/Ala (55% and 12%) and Glu/Asp (56% and 10%) (Suppementary Fig. S3). UMAMIT23-expressing cells never secreted more than 2.8-fold amino acids compared with control cells. Total detected amino acid secretion from yeast expressing the empty vector was 26 ± 3 µmol l^–1^ OD^–1^, which was significantly lower compared with the amino acids secreted by the yeast expressing UMAMIT23, UMAMIT24, and UMAMIT25 (35 ± 4, 59 ± 6, and 142 ± 9 µmol l^–1^ OD^–1^, respectively; *P*<0.05 according to *t*-test with *n*=4). The three UMAMITs expressed in yeast act as broad amino acid exporters, with a preference for Gln and/or Arg. When 22∆10α growth was tested on media containing amino acids as the sole nitrogen source, none of the three proteins could complement the transport defect and the growth of the yeast cells (data not shown), suggesting that none of them is able to mediate amino acid import at the concentrations tested. Because of the low activity of UMAMIT23, this gene was not included in further study. Uptake of radiolabeled Gln, Trp, His, Pro, or Met was measured in 22∆10α cells expressing UMAMIT24 or UMAMIT25. Neither cells showed a significant import of any of the amino acids tested compared with the cells expressing the empty vector (data not shown).

### UMAMIT24 and 25 are expressed in distinct cell types in developing seeds

**Fig. 2.**
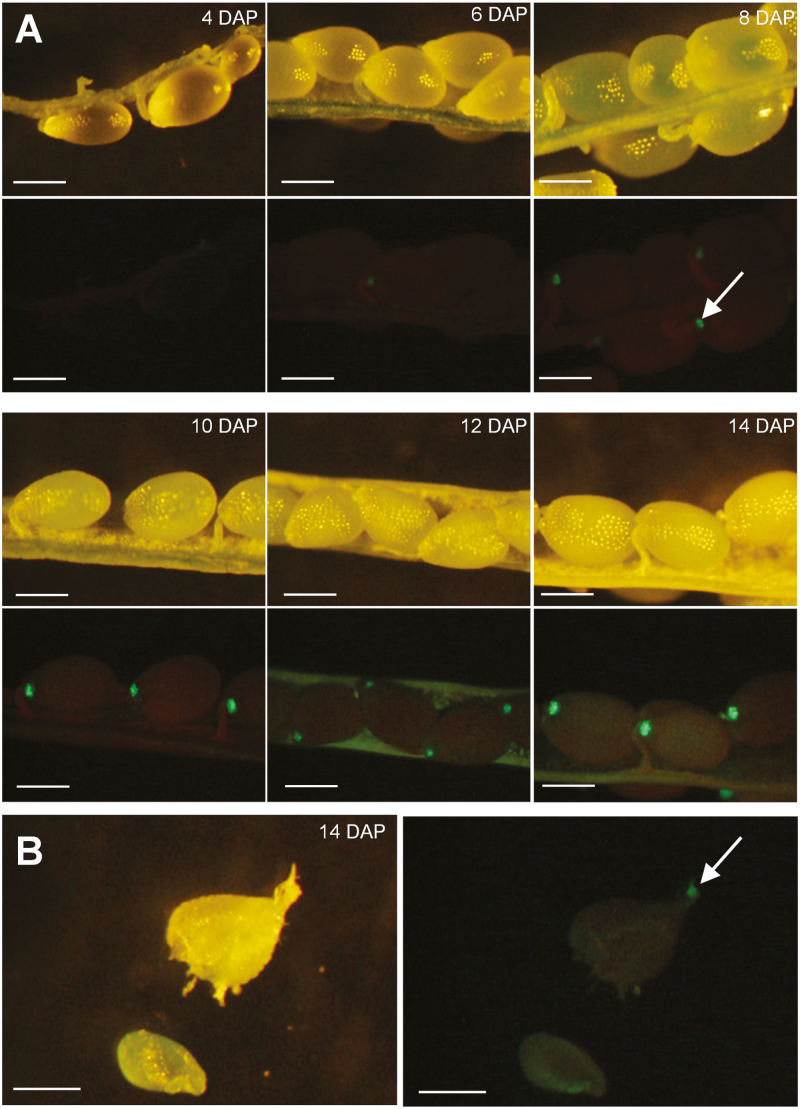
Localization of UMAMIT24 in Arabidopsis seeds. (A) Seeds from plants expressing UMAMIT24–GFP were observed with valves removed, under either bright light (top rows) or GFP excitation wavelength (bottom rows). (B) Dissected seeds revealing the embryo (bottom left of the picture) and the seed coat. The white arrowhead points to the chalazal seed coat. DAP, days after pollination.

**Fig. 3.**
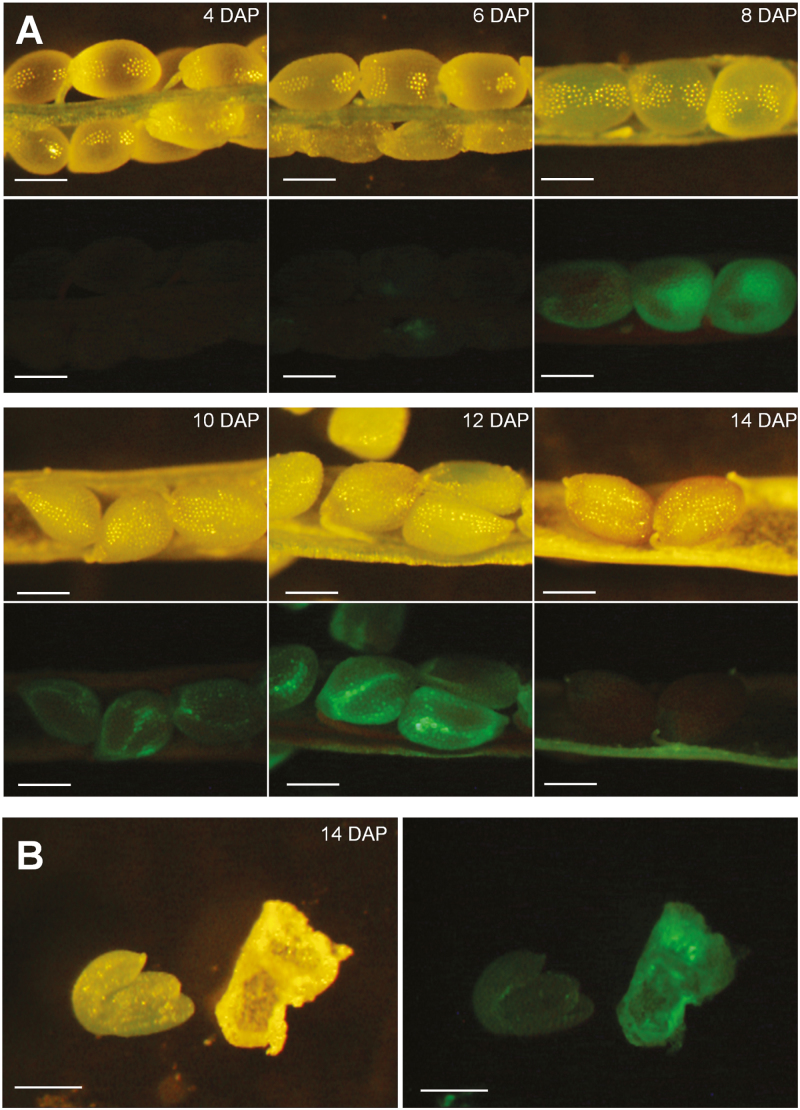
Localization of UMAMIT25 in Arabidopsis seeds. (A) Seeds from plants expressing UMAMIT25–GFP were observed with valves removed, under either bright light (top rows) or GFP excitation wavelength (bottom rows). (B) Dissected seeds revealing the embryo (bottom left of the picture) and the seed coat. DAP, days after pollination.

Publicly available microarray data suggested that both UMAMIT24 and UMAMIT25 are expressed in seed and silique tissues 6 DAP, and show low expression in vegetative tissues (Schmid *et al.*, 2005). To identify the cell types that express UMAMIT24 and 25 within the silique, UMAMIT24 or UMAMIT25 were tagged with GFP at the C-terminus and expressed under the control of their native promoter (*UMAMIT24promoter:UMAMIT24-GFP* and *UMAMIT25promoter:UMAMIT25-GFP*). Siliques were collected between 4 and 14 DAP, and GFP fluorescence was visualized in dissected siliques. UMAMIT24–GFP expression became visible at 6 DAP in the chalazal seed coat of developing seeds with little to no expression in the embryo, seed coat, or endosperm (Fig. 2A), whereas control plants did not show any fluorescence in the seeds (Supplementary Fig. S4A). The dissection of seeds at 14 DAP confirmed the exclusive expression of UMAMIT24 in the chalazal seed coat (Fig. 2B), with a weak fluorescence found in the embryo and pedicel, most probably due to autofluorescence (Supplementary Fig. S4B). In addition, UMAMIT24 expression was detected in the pericarp tissues, after 12 DAP (Fig. 2A). Confocal microscopy revealed that UMAMIT24–GFP expression in seeds was restricted to the chalazal seed coat (Supplementary Video S1). In Arabidopsis plants carrying the *UMAMIT25 promoter:UMAMIT25-GFP* construct, GFP fluorescence was visible 6 DAP in the endosperm of developing seeds (Fig. 3A), with little to no signal in the embryo (Fig. 3B). Similar to UMAMIT24, UMAMIT25–GFP expression was detected in the pericarp tissues 12 DAP. Confocal microscopy of UMAMIT25–GFP-expressing seeds at 12 DAP revealed that cells of the outer integument did not express UMAMIT25–GFP, whereas cells surrounding the embryo showed a strong expression of UMAMIT25–GFP (Supplementary Video S2), suggesting that in Arabidopsis seeds UMAMIT25 is exclusively expressed in the endosperm.

### Subcellular localization of UMAMIT24 and UMAMIT25

**Fig. 4.**
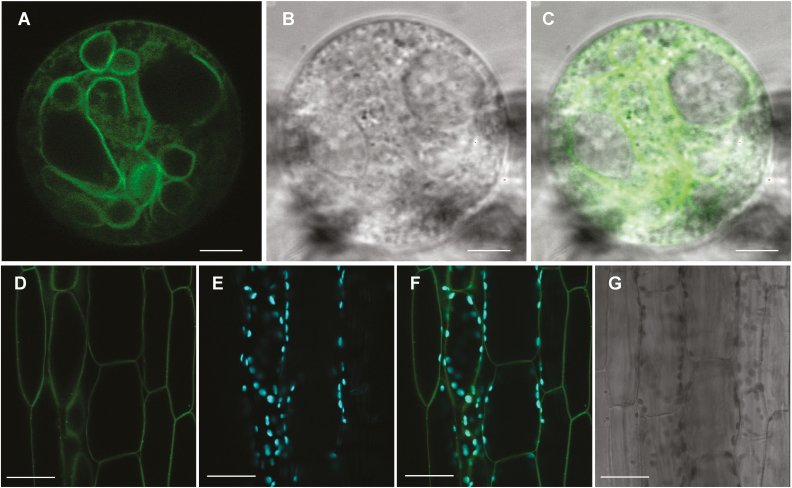
Subcellular localization of UMAMIT24–GFP and UMAMIT25–GFP. (A–C) Protoplasts from seeds expressing *UMAMIT24promoter:UMAMIT24-GFP*. (A) GFP, (B) bright field, (C) merged images. Scale bars are 5 µm. (D–G) Arabidopsis hypocotyl from 1-week-old seedlings stably expressing *35S:UMAMIT25-GFP.* (D) GFP, (E) chlorophyll, (F) merged, and (G) bright field. Scale bars are 20 µm.

Previously characterized UMAMIT members include transporters localized at the plasma membrane (Ladwig *et al.*, 2012; Müller *et al.*, 2015; Besnard *et al.*, 2016), tonoplast (Ranocha *et al.*, 2010; Ranocha *et al.*, 2013), and the ER membrane (Pan *et al.*, 2016). To determine the subcellular localization of UMAMIT24, developing seeds from plants expressing *UMAMIT24promoter:UMAMIT24-GFP* were imaged. Because chalazal seed coat cells are small and located several cell layers away from the seed surface, determining UMAMIT24–GFP subcellular localization using confocal microscopy within an intact seed was challenging; seeds expressing *UMAMIT24promoter:UMAMIT24-GFP*, cleared using 2,2'-thiodiethanol, revealed numerous round structures that could not be assigned to a specific cellular compartment (Supplementary Video S3). We thus prepared protoplasts from *UMAMIT24promoter:UMAMIT24-GFP*-expressing seeds to release GFP-positive cells from the seed. In these protoplasts, UMAMIT24–GFP signal was mainly found on the numerous tonoplasts (Fig. 4A–C; Supplementary Video S4), which have been previously observed for this cell type (Felker *et al.*, 1984: Millar *et al.*, 2015). On the other hand, when a construct harboring *CaMV35S promoter:UMAMIT24-GFP* was transiently expressed in Arabidopsis cotyledons or root cells, the GFP signal was localized to the ER membrane (Supplementary Figs S5A–F, S6). Similar results were obtained in cotyledons or in root cells using UMAMIT24 fused to the GFP tag at the N-terminus (Supplementary Fig. S5G–J). Our results indicate that UMAMIT24 localizes at the vacuolar membrane when expressed using the native promoter, and that the correct sorting of UMAMIT24 might require additional components that are lacking in the cotyledon and root cells. When UMAMIT25–GFP was overexpressed in Arabidopsis hypocotyl cells, the GFP signal was localized almost exclusively to the plasma membrane (Fig. 4D–G), which closely matched the localization of *UMAMIT25promoter:UMAMIT25-GFP* in the endosperm cells (Supplementary Fig. S7). These data suggest that the subcellular localization of UMAMIT24 and UMAMIT25 differs, with UMAMIT24 localizing mainly at the tonoplast and UMAMIT25 at the plasma membrane.

### Increase in UMAMIT24 and UMAMIT25 expression increases the seed yield

In order to understand UMAMIT24 and UMAMIT25 functions, a T-DNA insertion line for each gene has been isolated (*umamit24-1* and *umamit25-1*; Supplementary Fig. S1). qRT–PCR showed that the T-DNA insertion decreased the gene expression level in both cases, to ~4% of that of the WT for *umamit24-1*, and to undetectable levels for *umamit25-1* (Supplementary Table S1). In order to confirm that any phenotype was due to the lack of these UMAMIT genes, each T-DNA insertion line was complemented with an additional copy of the respective UMAMIT gene expressed under its native promoter (*umamit24-1/*UMAMIT24 and *umamit25-1/*UMAMIT25). Both lines expressed the introduced gene at a higher level than the WT (14.8 times for *umamit24-1/*UMAMIT24, 2.3 times for *umamit25-1/*UMAMIT25; Supplementary Table S1).

**Fig. 5.**
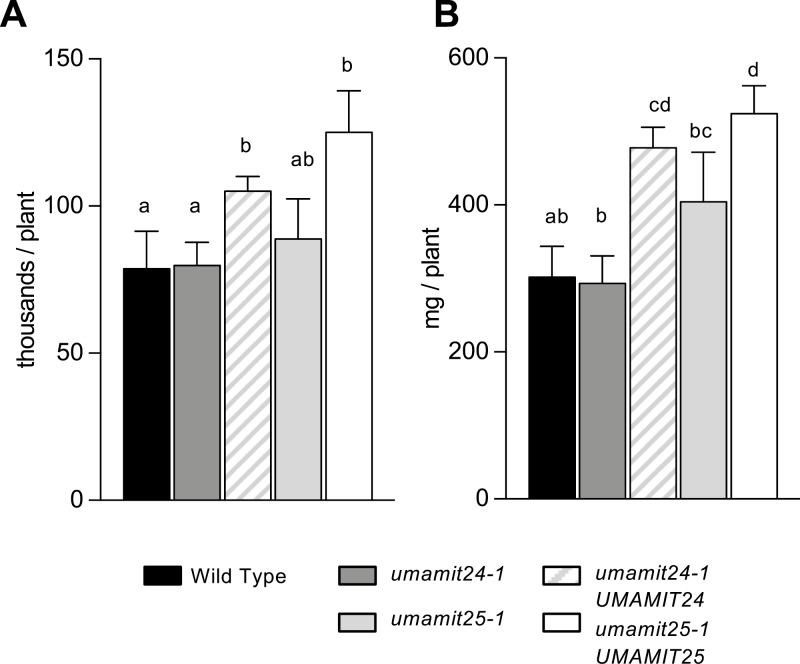
Seed yields of WT, *umamit*, and complemented lines. (A) Number of seeds produced per plant. (B) Mass of seeds per plant. Error bars correspond to the SD (*n*=4). Significant differences (*P*<0.05) are indicated by different letters according to one-way ANOVA in conjunction with Tukey’s test.

Since both UMAMIT24 and UMAMIT25 showed amino acid export activities in yeast and were found expressed in the developing seeds, seed number and mass of seeds produced per plant were measured for each of the mutants and complemented lines. No difference was observed between the two *umamit* knockout lines and the WT, while an increase in seed number and seed mass for both of the complemented lines was observed (Fig. 5). Weight for 100 seeds was slightly smaller for the *umamit25-1* and *umamit24-1/*UMAMIT24 lines compared with the WT (Supplementary Table S3). The percentages of carbon and nitrogen in seeds were analyzed in all the lines, and *umamit24-1/*UMAMIT24 seeds contained less nitrogen (4.82%) compared with all the other lines (between 5.1% and 5.4%; Supplementary Table S3). Finally, no significant differences were observed for the seed germination rate of all lines tested in long-day conditions (>90%, data not shown).

### The expression levels of UMAMIT24 and UMAMIT25 affect amino acid levels in the fruit tissues

**Fig. 6.**
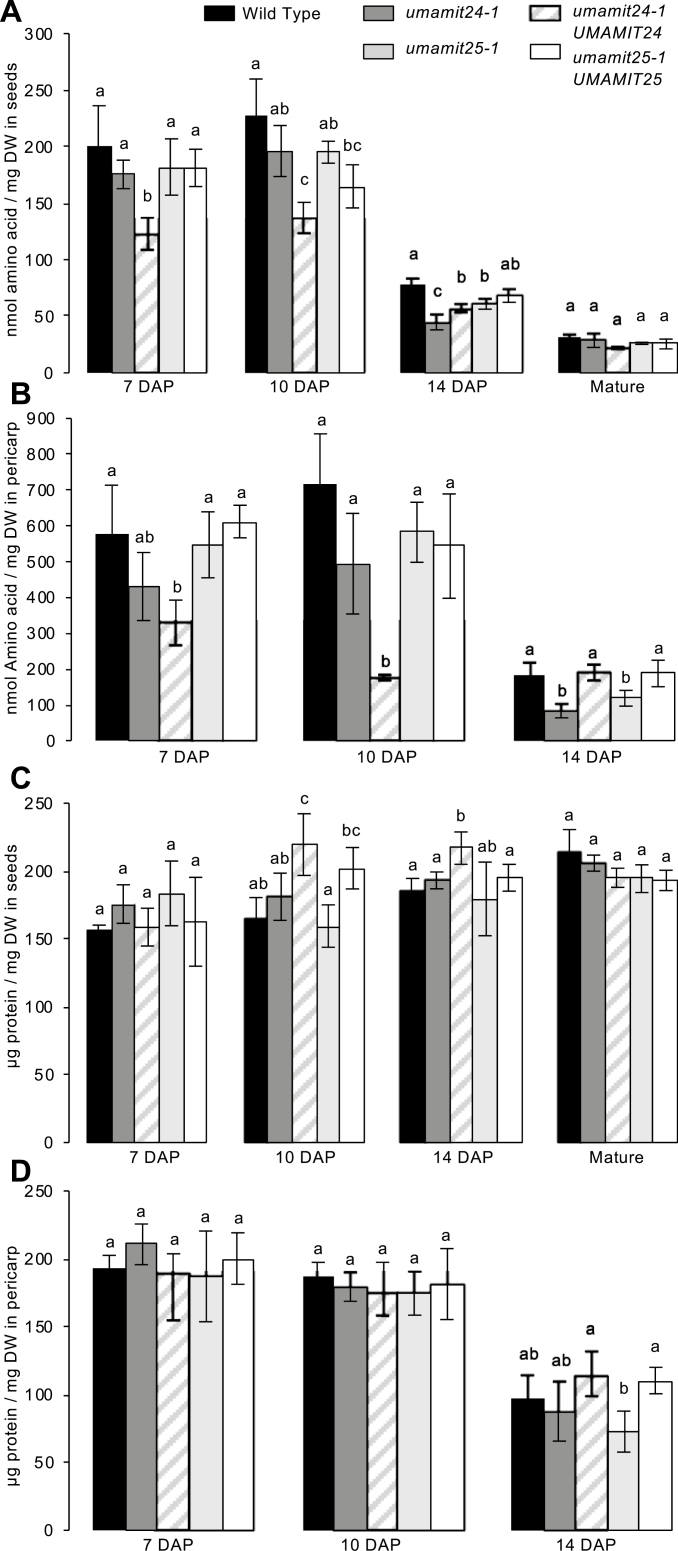
Protein and amino acid contents in seeds and pericarp tissue. Plants were grown side by side in soil under long-day conditions for 5 weeks. One biological replicate represents seeds or pericarp tissue from two siliques. Amino acid content in seeds (A) and pericarp tissue (B) or protein content in seeds (C) and pericarp tissue (D) are shown. Samples were harvested at 7, 10, and 14 DAP, or at the mature stage. Error bars correspond to the SD (*n*≥3 biological replicates). Significant differences (*P*<0.05) are indicated by different letters according to one-way ANOVA in conjunction with Tukey’s test. Contents of individual amino acids from the same data set are presented in Supplementary Tables S4 and S5.

To investigate further the potential roles of UMAMIT24 and UMAMIT25 in reproductive tissues, amino acid and protein contents were analyzed for the WT, the *umamit* knockout, and the complemented lines during seed development. To distinguish the effects of UMAMIT activities on amino acid accumulation in the silique from the effects on amino acid transfer to the seed, the developing seeds were separated from the pericarp (defined here as the silique minus the seeds; Supplementary Fig. S2). This experiment was performed at 7, 10, and 14 DAP, since UMAMIT24 and UMAMIT25 are both actively expressed during this period (Figs 2, 3). Estimated nitrogen in seed proteins accumulated quickly between 7 and 14 DAP, increasing nearly five times between the two time points. However, nitrogen in seed amino acids was only a small portion of total nitrogen at all time points (Supplementary Fig. S8A). Nitrogen in both proteins and amino acids decreased between 10 and 14 DAP in the pericarp tissue (Supplementary Fig. S8B). Further analysis revealed that in both *umamit24-1* and *umamit25-1* seeds, amino acid content at 14 DAP was significantly lower than that of the WT, which was partially restored in the complemented lines (Fig. 6A). These differences did not persist into the mature stage, at which none of the lines displayed any significant difference from the WT (Fig. 6A). Decreases in amino acid content at 14 DAP in both *umamit* mutants were also observed in the pericarp tissues, which were restored to WT levels in the complemented lines (Fig. 6B). Although seed protein content was unchanged between the WT and the *umamit* knockout lines at all the stages tested, seed protein content in the complemented line *umamit24-1*/UMAMIT24 was higher at 10 DAP (Fig. 6C). The seed protein content of this complemented line did not increase further after 10 DAP. In addition, the amino acid content at 10 DAP was lower in *umamit24-1/*UMAMIT24 seeds (Fig. 6A) and pericarp tissues (Fig. 6B) compared with the WT. A PCA revealed that the amino acid composition of *umamit24-1/*UMAMIT24 pericarp tissues at 10 DAP resembled that of other lines at 14 DAP, suggesting that maturation might be accelerated in the *umamit24-1/*UMAMIT24 complemented line (Supplementary Fig. S9). To confirm this hypothesis, we measured seed amino acid content at 14 DAP in an additional complemented line, *umamit24-1/*UMAMIT24-2, that expresses more UMAMIT24 than *umamit24-1/*UMAMIT24 (Supplementary Fig. S10A). The amino acid content of *umamit24-1/*UMAMIT24-2 seeds at 14 DAP was further reduced compared with *umamit24-1/*UMAMIT24 seeds (Supplementary Fig. S10B), suggesting that the phenotype observed in *umamit24-1/*UMAMIT24 correlates with UMAMIT24 expression levels, and that UMAMIT24 expression might change amino acid composition indirectly by accelerating seed development.

### Amino acid transfer to the seeds was reduced in the *umamit25-1 mutant*

**Fig. 7.**
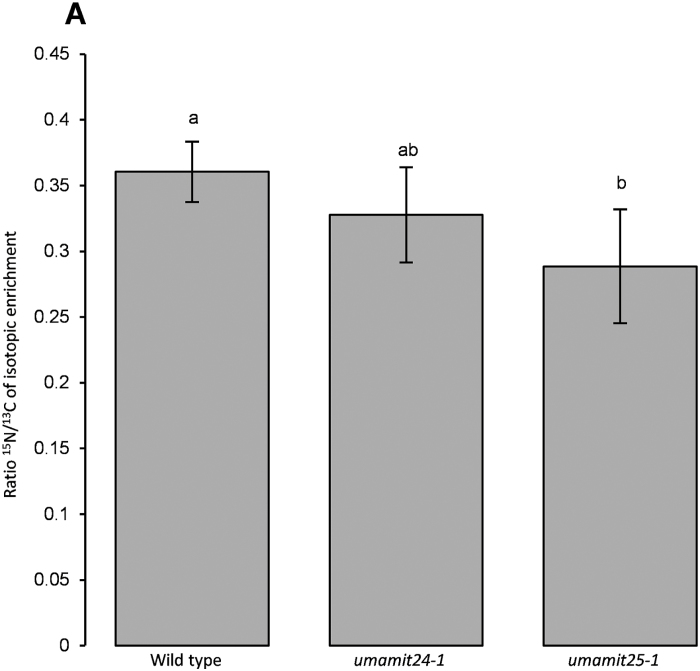
Glutamine and sucrose transfer assay in isolated siliques. ^15^N/^13^C ratio of isotopic excess found in seeds at 10 DAP. Siliques were excised 10 DAP, and transport was measured according to the experimental design presented in the Materials and methods. One biological replicate corresponds to three siliques worth of seeds coming from the same plant. ^15^N and ^13^C enrichment have been calculated against the non-labeled sample control. Error bars correspond to the SD (*n*≥3 biological replicates). Significant differences (*P*<0.05) are indicated by different letters according to one-way ANOVA in conjunction with Tukey’s test.

Amino acid content reduction in the *umamit* mutants could result either from a decrease in amino acid transport into the seeds or from an indirect effect related to a perturbation in amino acid metabolism. In order to measure amino acid transport to the seeds directly, the transfer of ^15^N-labeled Gln into the seeds was measured. In this assay, a solution containing [^15^N]Gln and [^13^C]sucrose was fed to the siliques for 4 h, followed by silique dissection and quantification of ^15^N and ^13^C enrichment in seeds. Siliques at 10 DAP were used for this experiment, since nitrogen loading in seeds is still ongoing at that time compared with 14 DAP (Supplementary Fig. S8; data not shown). The ^15^N/^13^C ratio was reduced in the *umamit25-1* plants compared with the WT (Fig. 7), suggesting that amino acid transport into the seeds is affected in the *umamit25-1* mutant. A similar, non-statistically significant trend was noted for the *umamit24-1* mutant.

## Discussion

### Proposed roles of UMAMIT24 and 25 during seed development

We have shown here that UMAMIT24 and UMAMIT25, previously uncharacterized members of the UMAMIT family, function as amino acid exporters when expressed in yeast (Fig. 1). These results are in good agreement with previous studies reporting amino acid export activities for other UMAMIT genes (Ladwig *et al.*, 2012; Müller *et al.*, 2015; Besnard *et al.*, 2016). In addition, we did not observe any import activity for either UMAMIT24 or UMAMIT25 for Gln, Trp, His, Pro, or Met under the conditions tested (data not shown).

UMAMIT24–GFP mainly localized to the tonoplast when expressed under its own promoter, whereas the signal was mostly found at the ER membrane when the fusion protein was transiently expressed in Arabidopsis cotyledons. This discrepancy could be an artifact caused in the transient expression system: exit of UMAMIT24 from the ER may require an accessory protein (Palacín and Kanai, 2004; Ruggiero *et al.*, 2008; Rosas-Santiago *et al.*, 2015, 2017) or a modifying protein (Popov-Čeleketić *et al.*, 2016), that are not expressed in a sufficient amount in the cotyledon cells. Together with previous studies that reported mislocalization of overexpressed proteins in non-native cell types (Okumoto *et al.*, 2004; Li *et al.*, 2005; Moore and Murphy, 2009), our result signifies the importance of using the native cell type for protein localization studies.

The expression of UMAMIT24 at the numerous vacuolar membranes was developmentally controlled, starting 6 DAP (Figs 3, 4; Supplementary Videos S3, S4). Ultrastructural studies of other angiosperm seeds revealed that the unloading domain cells during grain filling contain small and numerous vacuoles, suggesting that such a structural feature is conserved among plants (Felker *et al.*, 1984; Zheng and Wang, 2011; Millar *et al.*, 2015; Xurun *et al.*, 2015). The unloading domain in Arabidopsis becomes symplasmically isolated around stage 5 during embryogenesis (i.e. 6 DAP) (Stadler *et al.*, 2005), roughly corresponding to the onset of UMAMIT24 expression. Once such a transition happens, the unloading domain serves as the terminal point of nutrient transport from the maternal tissue, from which the solute needs to be exported to the apoplasm before subsequent uptake from the filial cells (Karmann *et al.*, 2018). Therefore, the unloading domain potentially represents a bottle-neck for solute transport, a notion supported by an enrichment of genes with transport function within this tissue (Becker *et al.*, 2014). Vacuoles in the unloading domains are suggested to serve as transient storage for solutes with lower mobility (i.e. metal ions), which might increase the solute storage capacity (Otegui *et al.*, 2002; Tauris *et al.*, 2009). Amino acids are readily mobile in the phloem and, so far, no evidence has been found for the requirement for transient amino acid storage in the vacuoles of the unloading tissue. On the other hand, amino acid content, velocity, and sugar/amino acid ratio in the source tissue phloem are known to fluctuate during the diurnal cycle (Troughton and Currie., 1977; Kallarackal *et al.*, 2012). It is tempting to hypothesize that the unloading domain of the seeds functions as a buffer to optimize the amino acid amount and composition delivered to the filial tissue, and that the delayed amino acid accumulation in *umamit24* mutant seeds is due to the decrease in amino acid storage capacity of vacuoles within the unloading domain cells.

Previous studies focused on UMAMIT11, UMAMIT14, and UMAMIT18 in Arabidopsis demonstrated that the corresponding protein–GFP fusions also localize in the chalazal seed coat during seed development, and that the knockout phenotype for these genes is also associated with a decrease (UMAMIT18) or increase (UMAMIT11 and UMAMIT14) of free amino acid content in siliques during seed development (Ladwig *et al.*, 2012; Müller *et al.*, 2015). However, these same studies showed that UMAMIT11, UMAMIT14, and UMAMIT18 localize at the plasma membrane, whereas our study shows that UMAMIT24 localizes in a vacuole-like structure (Fig. 4). These data suggest a different role for UMAMIT24 in the chalazal seed coat during seed development, since it is directed to a different membrane system.

UMAMIT25–GFP localized at the plasma membrane, and is expressed in the endosperm and the pericarp (Fig. 3). During Arabidopsis seed development, the endosperm transitions through three stages: syncytial, cellularization, and cellular (Becker *et al.*, 2014). During the cellularization stage, cell walls gradually develop around free nuclei to form endosperm cells. This transition happens 3–4 DAP, shortly before the onset of UMAMIT25 induction. UMAMIT25 could be involved in the amino acid export from mature endosperm cells, which consists of the last step of nutrient transport to the developing embryo, along with UMAMIT28, whose expression was detected first at the cellularizing endosperm (Müller *et al.*, 2015). Expression of UMAMIT24 and UMAMIT25 was also detected in the pericarp, but our analysis could not resolve in which tissue (Figs 2, 3). This suggests an additional role for these proteins, potentially in amino acid transport from the pericarp to the developing seeds.

### Loss of the expression of UMAMIT24 or UMAMIT25 affects amino acid transfer to the seed

Seed yields of *umamit24-1* and *umamit25-1* were not different from the WT, although a slight decrease in the size of *umamit25-1* seeds was observed (Fig. 5; Supplementary Table S2). Nevertheless, amino acid levels were lower in both seed and pericarp tissues in both of the *umamit* knockout lines at 14 DAP, corresponding to the late seed filling stage, and the difference was at least partially complemented by adding another copy of the genes (Fig. 6). At 10 DAP, the previous time point studied, the transfer of amino acids from the silique to the seed was reduced in the *umamit25-1* mutants (Fig. 7). Between 10 and 14 DAP, a rapid degradation of proteins is happening in the pericarp (Fig. 7; Supplementary Fig. S8), many of which are presumably transported to the seeds as amino acids. A study on two different canola varieties revealed that pod walls can serve as a reservoir for nitrogen for seeds, especially under low nitrogen conditions where up to 70% of the nitrogen found in seeds came from the pod walls (Girondé *et al.*, 2015). Similarly, we could hypothesize that during Arabidopsis embryogenesis, the nitrogen from the pericarp is remobilized in the form of amino acids, which are exported to the seeds by UMAMIT24 and UMAMIT25 starting at 12 DAP, the onset of their expression in the pericarp (Figs 2, 3). Based on these data, we hypothesize that the loss of UMAMIT24 and UMAMIT25 function decreases the transfer of amino acids from the pericarp to the seed, which would explain the decreased seed amino acid content at 14 DAP. The decrease in amino acid transfer from the pericarp could create a negative feedback on protein degradation and/or amino acid transfer from the mother tissue, hence decreasing the free amino acid content in the pericarp. This feedback would be reminiscent of the effect observed in the shoot to root transfer of amino acids in the *umamit14* and *18* loss-of-function plants (Besnard *et al.*, 2016). The difference in amino acid content, however, did not translate into a decrease in mature seed protein content, unlike the previous studies in which amino acid loading capacity at the source and seeds positively correlated with seed protein content (Zhang *et al.*, 2010, 2015; Santiago and Tegeder, 2016, 2017). In *umamit24 and 25* knockout plants, amino acid phloem loading capacities at the source have not been altered; therefore, the amino acid deficit during the seed growth might have been compensated by a slightly longer filling period. Indeed, previous studies have shown that the duration of seed filling is altered in different conditions, and that seed nitrogen filling terminates when the mobile nitrogen pool is exhausted at the source (Egli *et al.*, 1985; Munier-Jolain *et al.*, 1996; Girondé *et al.*, 2015). Similar results have been reported for seed phosphorus filling, where loss of function of seed phosphate transporters decreases phosphorus content in developing embryo but not in mature seeds (Vogiatzaki *et al.*, 2017).

**Fig. 8.**
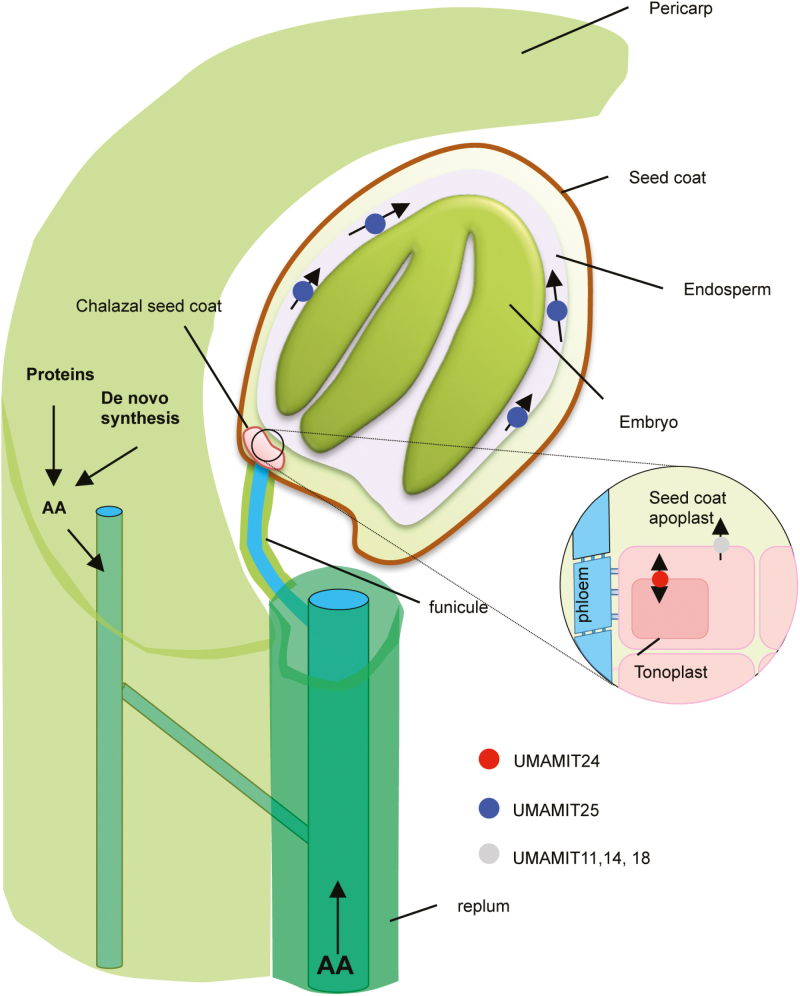
Model of amino acid transport into the seed by UMAMIT24 and UMAMIT25. Amino acids are delivered from the mother plant to the growing embryo through the funicule, then unloaded from the chalazal seed coat. UMAMIT24 localizes at numerous vacuoles in the chalazal seed coat and is involved in this process, possibly acting as a transporter for temporary storage of amino acids in this tissue. Once out of the chalazal, seed coat amino acids are transported radially through the integument cells then delivered to the endosperm, and finally to the embryo. UMAMIT25, expressed in the endosperm cells, unloads amino acids out of those cells which then become available for import by the embryo. Loss of function of UMAMIT25 reduces the amino acid unloading process, and decreases amino acid transfer from the rest of the plant body to the growing embryo.

The roles of UMAMIT24 and UMAMIT25 are summarized in the model presented in Fig. 8. Amino acids delivered by the phloem in the unloading zone could be transported by UMAMIT24 in the vacuoles of the chalazal seed coat cells and temporarily stored before being transported to the filial tissue. On the other hand, UMAMIT25 could be involved in the unloading of amino acids from the endosperm. The lack of these activities results in a decreased amino acid unloading to the seed tissue, which is reflected in the decrease in seed amino acid content at 14 DAP. The decrease in the unloading also results in the slower amino acid transfer from the mother tissue, which affects the amino acid content in the pericarp tissue. The extent to which the lack of UMAMIT24 and 25 activity in the pericarp is contributing to this effect remains to be elucidated.

### Increased expression of UMAMIT24 and UMAMIT25 increases yields

Both of the complemented lines used in this study showed an increased expression of UMAMIT genes in the seeds compared with the WT (Supplementary Table S1). We have previously observed an increased expression of a gene from an additional copy of a gene within a T-DNA compared with the expression of the gene in the WT: ~50 times for a UMAMIT (Besnard *et al.*, 2016), and an 8-fold increase for the *LOG2* gene, when a 8 kb DNA fragment corresponding to 4 kbp upstream and 2 kb downstream from the transcribed region was used to complement the *log2-1* mutant (Pratelli *et al.*, 2012; GP, unpublished data). This phenomenon could be caused by a bias for a stronger expression of the marker gene during the selection process (Kim and Gelvin, 2007), the lack of regulatory elements in the regions excluded from the constructs used to generate the complemented lines (Singer *et al.*, 2012; Shen *et al.*, 2017), or more than one T-DNA inserted at the insertion site (Robinson *et al.*, 2009). Amino acid contents of both seeds and pericarp tissues at earlier time points were decreased in the UMAMIT24 complemented line compared with the WT, but not in the UMAMIT25 complemented line. A PCA revealed that the amino acid composition of the pericarp tissues of the UMAMIT24 complemented line at 10 DAP resembled that of other lines at 14 DAP, suggesting that the pericarp maturation is accelerated in this line (Supplementary Fig. S9). This effect could be due to the overexpression of *UMAMIT24* (>14-fold compared with the WT; Supplementary Table S1), which, based on the proposed role of the protein in seed filling (see above), would increase amino acid fluxes coming from the pericarp tissues to the seeds. An increased activity of UMAMIT24 or UMAMIT25 resulted in an increase in total seed number and seed mass produced per plant (Fig. 5), while seed protein and amino acid content per dry weight were unchanged in the mature seeds (Fig. 6). Although the exact mechanism for the increase in seed yield in both of the complemented lines is unclear, this result offers the possibility of utilizing an increased amino acid export capacity to increase seed yield. In this context, it is important to note that UMAMIT24 and UMAMIT25 are expressed almost exclusively in the seed and pericarp tissues; hence, it is less likely that an increase in their activities would cause a detrimental effect in other tissues. Several recent studies have shown that changes in the activity of amino acid importers represent an effective way to increase seed yields in multiple plant species (Zhang *et al.*, 2010, 2015; Perchlik and Tegeder, 2017). A similar strategy using amino acid exporters might be effective in increasing crop yield in other plant species, if UMAMITs that are exclusively expressed in these species’ seed/fruit tissues can be discovered.

## Supplementary data

Supplementary data are available at JXB online.

Fig. S1. Location of the T-DNA insertion in *umamit24-1* and *umamit25-1*.

Fig. S2. Experimental procedure for the acquisition of 7-, 10-, or 14-day-old siliques.

Fig. S3. Relative abundance of amino acids secreted by yeast expressing UMAMIT24 and UMAMIT25

Fig. S4. Wild-type fluorescence of Arabidopsis silique.

Fig. S5. Transient expression of GFP-tagged UMAMIT24 and an ER marker (HDEL–mCherry) in Arabidopsis cotyledons.

Fig. S6. Ectopic expression of 35S:UMAMIT24–GFP in Arabidopsis roots.

Fig. S7. Localization of UMAMIT25–GFP in the endosperm cells

Fig. S8. Accumulation of nitrogen in proteins and amino acids in seed and pericarp tissues.

Fig. S9. PCAs of amino acid content in the envelope tissue.

Fig. S10. *UMAMIT24* mRNA accumulation in siliques and amino acid content in seeds of *umamit24*/UMAMIT24 complemented lines at 14 DAP

Table S1. *UMAMIT24* and *UMAMIT25* mRNA expression levels in 14-day-old developing seeds.

Table S2. Primers used for cloning and qRT–PCR.

Table S3. Plant and seed biomass obtained on plants at maturity.

Table S4. Amino acid content in 7-, 10-, and 14-day-old and mature seeds.

Table S5. Amino acid content in 7-, 10-, and 14-day-old pericarp tissues.

Video S1. UMAMIT24–GFP expression in the seed.

Video S2. UMAMIT25–GFP expression in the seed.

Video S3. UMAMIT24–GFP expression in chalazal seed coat.

Video S4. A protoplast prepared from chalazal seed coat cells expressing UMAMIT24–GFP.

Supplementary Video S1

Supplementary Video S2

Supplementary Video S3

Supplementary Video S4

Supplementary Figures and Tables
