# Supplementary material for: Arabidopsis UMAMIT24 and 25 are amino acid exporters involved in seed loading
Source: J Exp Bot. 2018 Aug 24;69(21):5221–32. doi: 10.1093/jxb/ery302 (PMC6184519; doi:10.1093/jxb/ery302)
Supplement: Supplementary Figures and Tables [file ery302_suppl_supplementary_figures_tables.pdf]

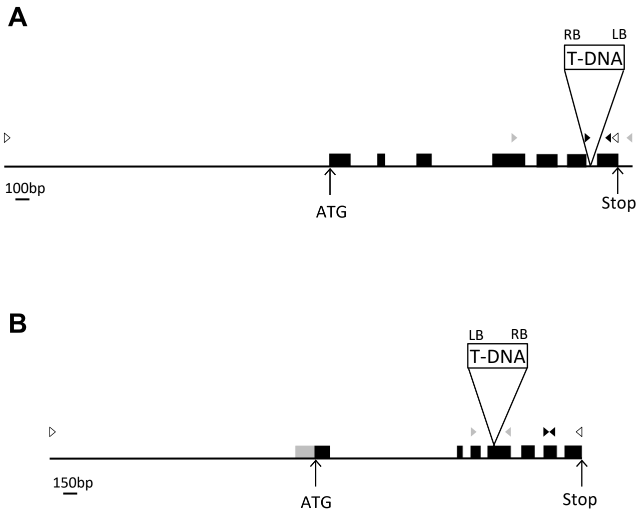

Figure S1. Location of the T-DNA insertion in *umamit24-1* and *umamit25-1*. (A) and (B) location of the T-DNA insertion in *umamit24-1* and *umamit25-1,* respectively*.* Grey and black boxes represent untranslated and translated regions of exons, respectively*.* Closed arrowheads represent the positions of forward and reverse primers used for qRT-PCR. The forward primer used for *UMAMIT24* qRT-PCR amplification was specific to the cDNA at the transition exon 6 – exon 7. Open arrowheads represent the positions of the primers used for creation of the complemented line. Grey arrowheads represent the position of the primers used for genotyping the T-DNA insertion.

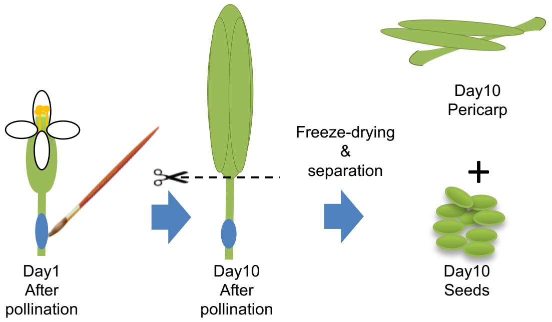

Figure S2. Acquisition of 7, 10 or 14 day-old silique experimental procedure. The example above is given for day 10 after pollination. A newly opened flower was considered day 1 after pollination and its pedicel was painted. Nine days later (day 10 after pollination), the pedicel was removed, the siliques were freeze-dried and the seeds were separated from the pericarp (defined here as the silique minus the seeds). In case of the glutamine and sucrose transfer assay in isolated siliques, the silique on top of and underneath the painted silique were used to avoid potential interference caused by the paint. All labeling and harvesting happened during early afternoon.

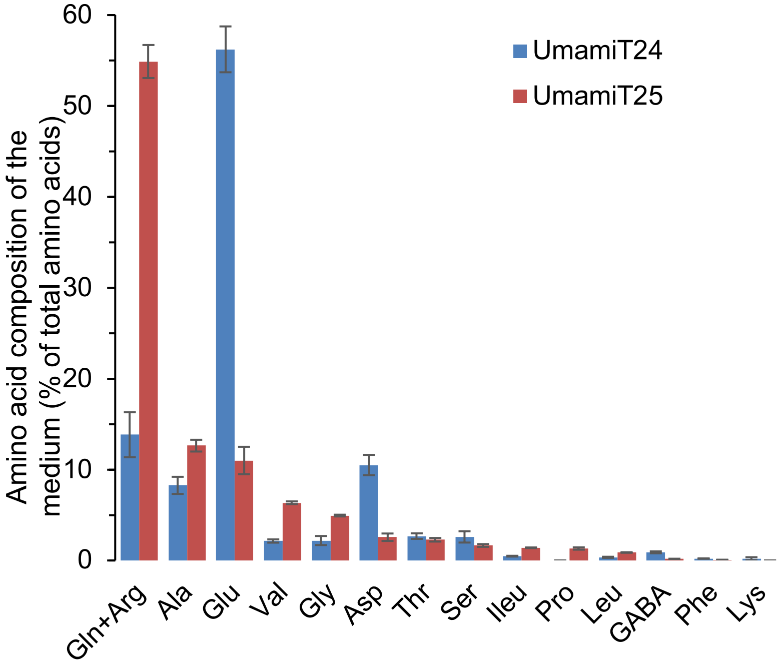

Figure S3. Relative abundance of amino acids secreted by yeast expressing UMAMIT24 and UMAMIT25. Amino acid contents represented in Figure 1 were used. The average of content obtained for the empty plasmid was subtracted to the content of each biological replicates of UMAMIT14 and UMAMIT18. Each value, which represents amino acid secretion above the background level, was divided by the sum of these normalized values, and expressed as a percentage. Error bars correspond to the standard deviation with n=4. GABA: gamma-amino-butyric acid.

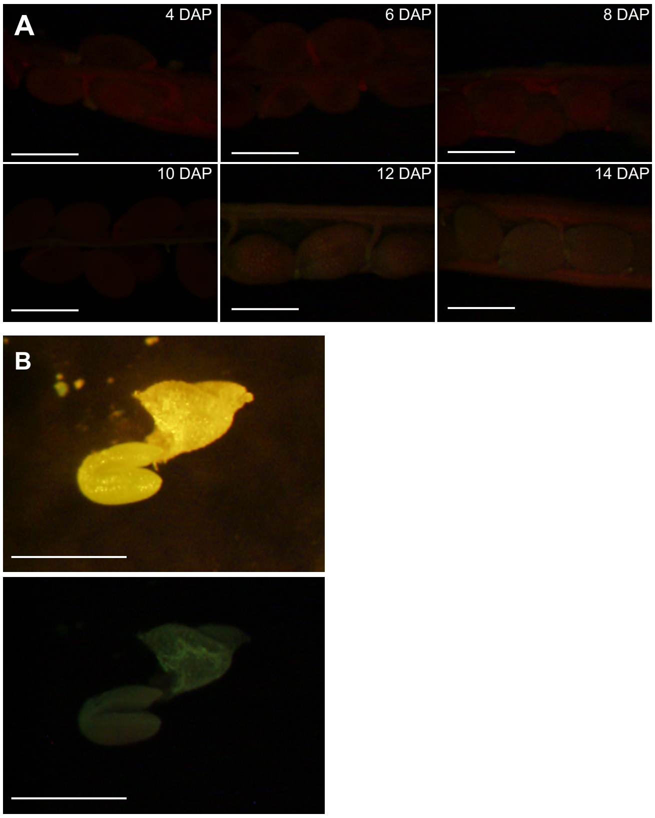

Figure S4. Wild type fluorescence of Arabidopsis silique. A: Siliques with valves removed under GFP-excitation wavelength. B: Dissected seeds revealing the embryo (bottom left of the picture) and the seed coat. For panel B, seeds were observed under bright light (top row) or under GFP-excitation wavelength (bottom row). DAP: day after pollination. Scale bars are 200 µm.

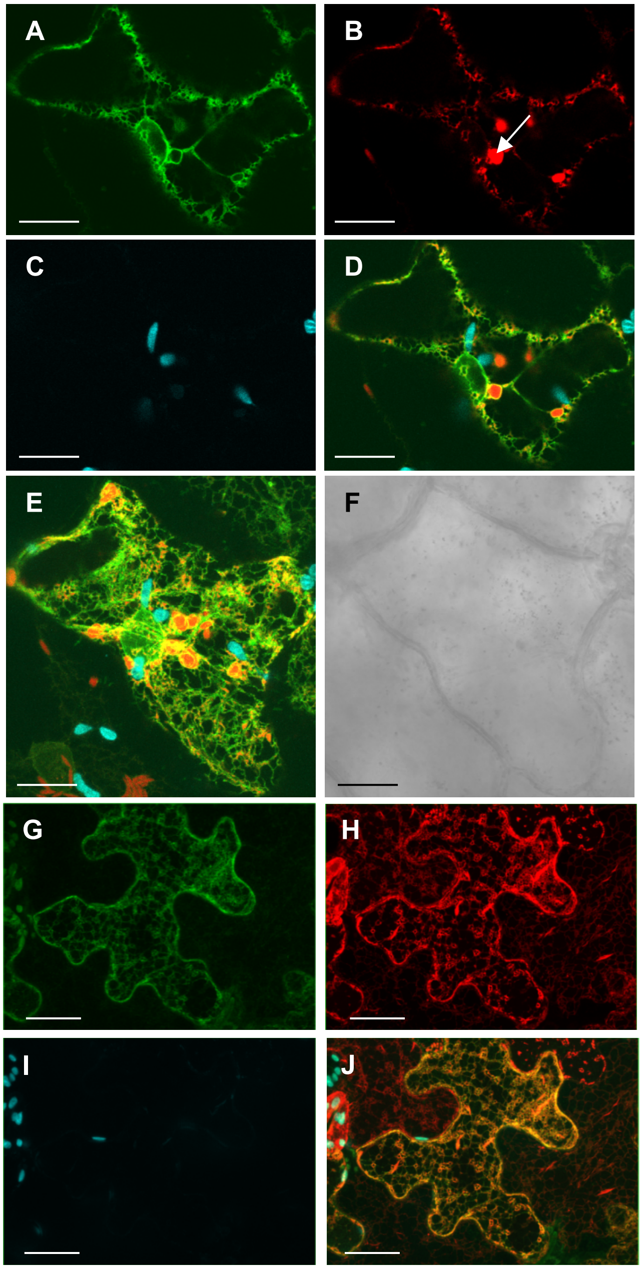

Figure S5. Transient expression of GFP-tagged UMAMIT24 and an ER marker (HDEL-mCherry) in Arabidopsis cotyledons. (A-F) 35S:UMAMIT-GFP in Arabidopsis cotyledons. (A-F) Cotyledons co-transformed with *35S:UMAMIT24-GFP* and *HDEL-mCherry*. (A) GFP, (B) mCherry, (C) chlorophyll, (D) merged, (E) Z-Stack of the cell displayed in A-D and (F) bright field. (G-J) Cotyledons co-transformed with *35S:GFP-UMAMIT24* and *HDEL-mCherry* (A) GFP, (B) HDEL-mCherry, (C) chlorophyll and (D) merged. Scale bars are 20 µm.

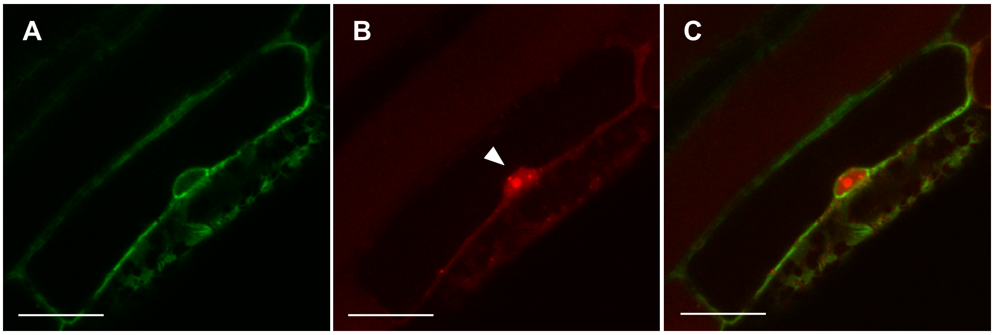

Figure S6. Ectopic expression of 35S:UMAMIT24-GFP in Arabidopsis roots. (A) GFP, (B) Syto82® labelling the DNA and (C) merged. White arrowhead points to the nucleus. Scale bars are 20 μm.

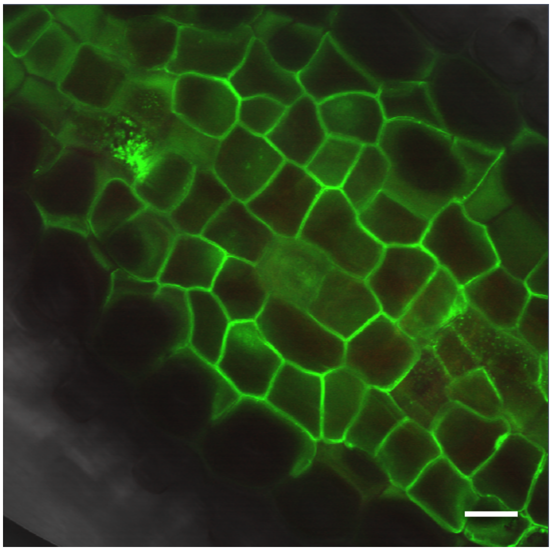

Figure S7. Localization of UMAMIT25-GFP in the endosperm cells. Cell boundaries are clearly labeled, suggesting the plasma membrane localization of the fusion protein. Scale bar: 20 µm.

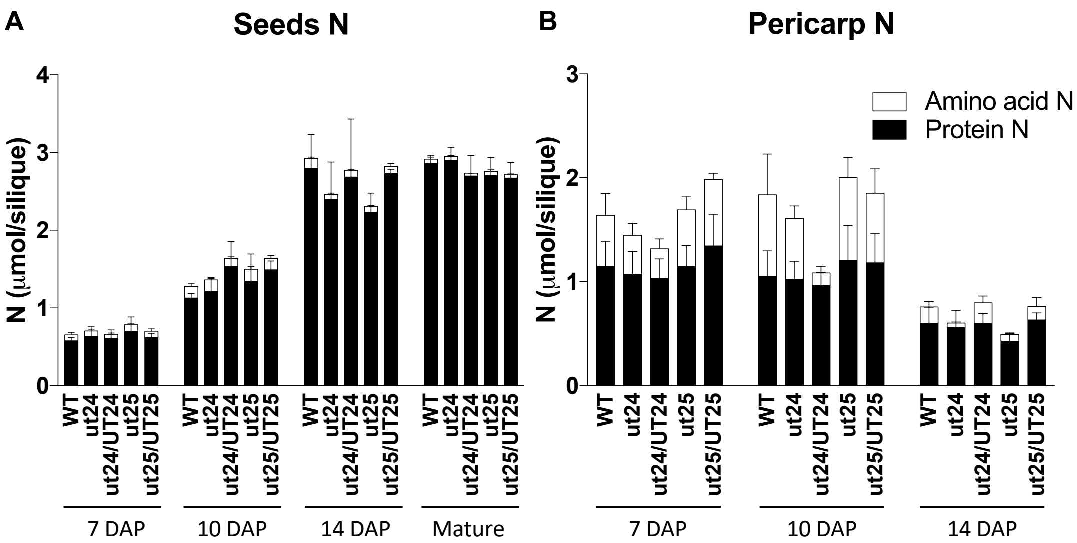

Figure S8. Accumulation of nitrogen in proteins and amino acids in seed and pericarp tissues. (A) Nitrogen in proteins and amino acids in seeds at 7, 10, 14 DAP and mature seeds. (B) Nitrogen in proteins and amino acids in the pericarp tissues at 7, 10 and 14 DAP. Nitrogen in seed and pericarp proteins was estimated as described in the material and methods. Amino acid contents were derived from the data represented in Table S4 and S5. Closed and open bars represent nitrogen from proteins and amino acids, respectively. WT, wild type; ut24, *umamit24-1*; ut24/UT24, *umamit24-1*/UMAMIT24; ut25, *umamit25-1*; and ut25/UT25, *umamit25-1*/UMAMIT25 lines.

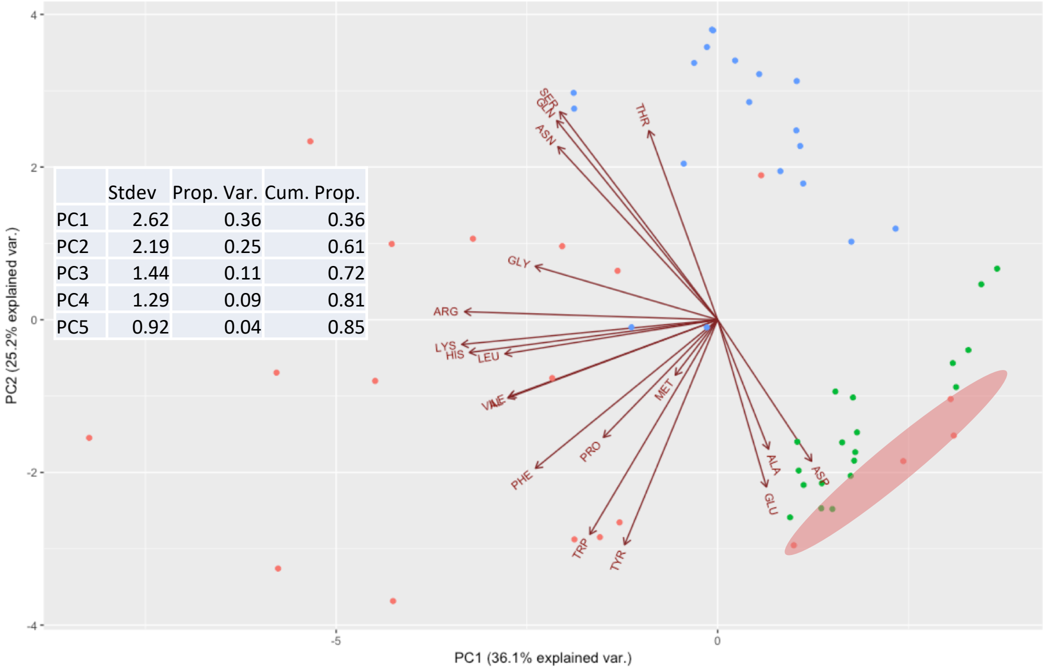

	Stdev	Prop. Var.	Cum. Prop.
PC1	2.62	0.36	0.36
PC2	2.19	0.25	0.61
PC3	1.44	0.11	0.72
PC4	1.29	0.09	0.81
PC5	0.92	0.04	0.85

Figure S9. PCA analysis of amino acid content in the pericarp tissue. Amino acid content of pericarp tissue (presented in Table S5) has been analyzed. The data points at 7, 10, and 14 DAP are marked with blue, red and green, respectively. The four points representing 10 DAP *umamit24-1/*UMAMIT24 are marked with an ellipse (probability = 0.68). Inset: the loadings of PCA analysis. Stdev, standard deviation; Prop. Var, proportion of variance; Cum.Prop, cumulative proportion.

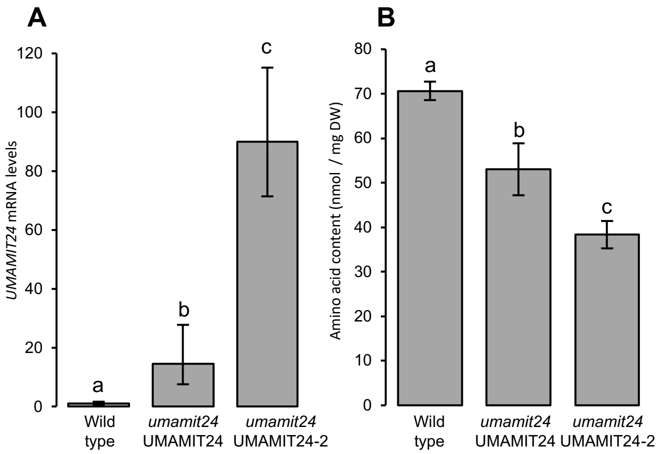

Figure S10. *UMAMIT24* mRNA accumulation in siliques and amino acid content in seeds of *umamit24* / UMAMIT24 complemented lines at 14 DAP. (A): *UMAMIT24* mRNA expression levels in 14 DAP developing siliques. *UMAMIT24* mRNA levels obtained by RT-qPCR have been normalized against Actin 8 and are relative to wild type expression. (B): amino acid content in seeds at 14 DAP is shown. Means and standard deviations are displayed (n=3 biological replicates). Significant differences ($p<0.05$) are indicated by different letters according to one way ANOVA in conjunction with Tukey's test.

Table S1. *UMAMIT24* and *UMAMIT25* mRNA expression levels in 14 day-old developing seeds. Means and standard deviations are displayed (n=3). Significant differences ($p<0.05$) are indicated by different letters according to one way ANOVA in conjunction with Tukey's test. N.D. no amplification was detected. *UMAMIT24* and *UMAMIT25* mRNA levels obtained by RT-qPCR have been normalized against Actin 8 and are relative to wild type expression.

	UMAMIT24		*UMAMIT25*
Wild type	1<br>± 0.13 a	Wild type	1<br>± 0.06 a
umamit24-1	0.043<br>± 0.03 b	*umamit25-1*	n.d.
umamit24-1<br>UMAMIT24	14.79<br>± 7.5 c	*umamit25-1*<br>UMAMIT25	2.30<br>±0.032 b

Table S2. Primers used for cloning and qRT-PCR. Primer sequences are given from 5' to 3'. Underlined bases were added for the creation of *attB1* and *attB2* in forward and reverse primers, respectively.

**Name**	**Sequence**
UMAMIT24 promoter (Forward)	GGACAAGTTTGTACAAAAAAGCAGGCTTCAGTTCATCTTCTGCAGAACGACCAA
UMAMIT24 end of gDNA (Reverse)	GGACCACTTTGTACAAGAAAGCTGGGTCGGGGACATCTCTATTTACTGATGAAAGATT
UMAMIT25 promoter (Forward)	GGACAAGTTTGTACAAAAAAGCAGGCTTCGCTCGGGATTTGAAATGGAGGA
UMAMIT25 end of gDNA (Reverse)	GGACCACTTTGTACAAGAAAGCTGGGTCAGGCGATGTAGACCTTGTGGAACC
UMAMIT23 cDNA expression in yeast (Forward)	GACAAGTTTGTACAAAAAAGCAGGCTCAGAAATGAAAGATATAACGGCAATG
UMAMIT23 cDNA expression in yeast (Reverse)	GACCACTTTGTACAAGAAAGCTGGGTACYAAGGGACATTTGTACTTAATGTTGG
UMAMIT24 cDNA expression in yeast (Forward)	GACAAGTTTGTACAAAAAAGCAGGCTCAGGAGAAATGAAGAGTGTAGTTGCA
UMAMIT24 cDNA expression in yeast (Reverse)	GACCACTTTGTACAAGAAAGCTGGGTACYAGGGGACATCTCTATTTACTGATGAA
UMAMIT25 cDNA expression in yeast (Forward)	GACAAGTTTGTACAAAAAAGCAGGCTCAGAGATGGCTAAATCAGATATGTTGC
UMAMIT25 cDNA expression in yeast (Reverse)	GACCACTTTGTACAAGAAAGCTGGGTACYAAGGCGATGTAGACCTTGTGG
Actin 8 qRT-PCR (Forward)	GTGTCTGGATTGGTGGTTCTATCC
Actin 8 qRT-PCR (Reverse)	GCCTTAGAGATCCACATCTGCTG
UMAMIT24 qRT-PCR (Forward)	TTTGGGAAGTATAATCGGTGCC
UMAMIT25 qRT-PCR (Reverse)	AAAGATTGCCAAGGTCCAGTTCT
UMAMIT25 qRT-PCR (Forward)	TGGGCTATGCAGAGGAAAGGTC
UMAMIT25 qRT-PCR (Reverse)	CCACAAGTGCTGATCCCATAAACG

Table S3. Plant and seed biomass obtained on plants at maturity. Dead biomass includes the primary root and all aerial parts, except the seeds. Means and standard deviations are displayed with n=4. Significant differences (p<0.05) are indicated by different letters according to one way ANOVA in conjunction with Tukey's test.

	Dead Biomass (g)	Seed number (thousands)	Seed mass (mg)	Weight of 100 seeds (mg)	Seed %C	Seed %N
Wild type	1.07 ± 0.07 a	78.7 ± 12.7 a	301.6 ± 42.3 ab	2.60 ± 0.11 a	55.9 ± 0.72 a	5.40 ± 0.17 a
umamit24-1	1.1 ± 0.17 a	79.8 ± 7.91 a	293.3 ± 37.5 b	2.74 ± 0.39 ab	56.3 ± 0.73 a	5.34 ± 0.23 a
umamit24-1 UMAMIT24	1.01 ± 0.01 a	105 ± 5.07 b	477.5 ± 28.2 cd	2.21 ± 0.08 b	56.3 ± 0.42 a	4.82 ± 0.02 b
umamit25-1	1.04 ± 0.03 a	88.8 ± 13.6 ab	404 ± 67.7 bc	2.20 ± 0.07 b	55.9 ± 0.66 a	5.13 ± 0.18 a
umamit25-1 UMAMIT25	1.22 ± 0.13 a	125 ± 14.2 b	524 ± 38.1 d	2.38 ± 0.17 ab	56.5 ± 0.80 a	5.10 ± 0.20 a

Table S4. Amino acid content in 7, 10, 14 day-old and mature seeds. Values are expressed in nmol per µg of dry weight. Each sample corresponds to two siliques worth of seeds coming from the same plant. Means and standard deviations are displayed with n=3. Significant differences ($p<0.05$) are indicated by different letter according to one way ANOVA in conjunction with Tukey's test.

Day 7	ASP	GLU	ASN	SER	GLN	HIS	GLY	THR	ALA	ARG	TYR	VAL	MET	TRP	PHE	ILE	LEU	LYS	PRO	Sum
Wild type	5.43 ± 0.74 a	22.8 ± 1.36 a	7.85 ± 2.08 a	48.6 ± 8.28 a	30.7 ± 12.2 a	1.47 ± 0.18 a	13.4 ± 7.55 a	7.77 ± 1.66 a	9.09 ± 0.71 a	6.71 ± 1.49 a	1.94 ± 0.13 a	5.78 ± 0.17 a	13 ± 1.17 a	0.76 ± 0.12 a	3.28 ± 0.05 a	4.08 ± 0.26 a	6.68 ± 0.21 a	5.09 ± 0.02 a	5.65 ± 1.57 a	200 ± 35.8 a
umamit24-1	6.14 ± 0.94 a	22.3 ± 2.99 a	11.2 ± 4.45 a	37.8 ± 7.92 ab	32.8 ± 9.05 a	0.89 ± 0.28 bc	5.95 ± 1.77 ab	8.22 ± 1.94 a	8.65 ± 1.59 a	6.65 ± 1.47 a	1.67 ± 0.5 a	5.61 ± 1.38 a	5.02 ± 0.86 b	0.5 ± 0.17 ab	2.68 ± 0.74 ab	3.37 ± 0.89 ab	6.2 ± 1.73 a	2.47 ± 1.28 b	6.96 ± 1.76 a	175 ± 12.6 a
umamit24-1 UMAMIT24	5.66 ± 0.71 a	20.7 ± 1.34 a	8.09 ± 1.35 a	21.7 ± 4.17 b	13.9 ± 1.97 a	0.63 ± 0.15 c	3.49 ± 0.36 b	7 ± 1.52 a	6.7 ± 0.88 a	3.1 ± 1.09 b	1.26 ± 0.24 a	4.06 ± 0.84 a	7.04 ± 1.39 b	0.4 ± 0.08 b	2 ± 0.32 b	2.42 ± 0.59 b	4.47 ± 0.85 a	2.31 ± 0.77 b	7.77 ± 3.16 a	122 ± 14.3 b
umamit25-1	6.35 ± 0.52 a	23.3 ± 4.8 a	8.16 ± 1.03 a	41.8 ± 14.6 a	38.4 ± 17.1 a	1.08 ± 0.18 ab	6.99 ± 3.48 ab	6.17 ± 1.14 a	8.56 ± 0.77 a	5.58 ± 0.94 ab	1.71 ± 0.38 a	5.99 ± 0.97 a	6.01 ± 1.27 b	0.52 ± 0.1 ab	2.62 ± 0.64 ab	3.42 ± 0.71 ab	6.31 ± 1.26 a	2.32 ± 1.01 b	6.45 ± 4.72 a	182 ± 24.9 a
umamit25-1 UMAMIT25	5.86 ± 0.42 a	22.4 ± 2.7 a	10.5 ± 3.58 a	36.5 ± 5.3 ab	43.3 ± 19.2 a	1.05 ± 0.13 abc	5.69 ± 0.72 ab	6.78 ± 0.92 a	8.53 ± 0.66 a	5.03 ± 0.86 ab	1.7 ± 0.36 a	5.55 ± 0.68 a	7.24 ± 1.31 b	0.51 ± 0.09 ab	2.69 ± 0.47 ab	3.26 ± 0.39 ab	6.1 ± 0.95 a	3.11 ± 0.77 ab	5.19 ± 1.77 a	181 ± 16.4 a

Day 10	ASP	GLU	ASN	SER	GLN	HIS	GLY	THR	ALA	ARG	TYR	VAL	MET	TRP	PHE	ILE	LEU	LYS	PRO	Sum
Wild Type	5.25 ± 1.06 a	21 ± 2.02 a	12.2 ± 3.37 a	73.2 ± 33.9 a	25.7 ± 3.85 a	1.11 ± 0.03 a	8.11 ± 1.99 a	7.87 ± 0.64 a	11.4 ± 2.72 a	10.9 ± 1.57 a	3.13 ± 0.71 a	8.08 ± 1.57 a	7.81 ± 0.29 a	0.85 ± 0.25 a	5.65 ± 1.38 a	6.27 ± 1.39 a	12.2 ± 2.93 a	7.77 ± 1.61 a	5.26 ± 1.48 a	234 ± 36.2 a
umamit24-1	5.34 ± 0.7 a	17.9 ± 4.97 a	24.2 ± 10.7 a	43 ± 14.1 ab	30.1 ± 9.39 a	1.18 ± 0.39 a	5.09 ± 2.36 ab	6.79 ± 0.9 a	8.42 ± 3.57 a	9.42 ± 2.93 a	2.17 ± 1.52 a	5.6 ± 3.14 a	7.58 ± 1.96 a	0.5 ± 0.33 a	4 ± 2.82 a	4.25 ± 2.74 a	8.45 ± 6.04 a	7.04 ± 3.71 a	4.89 ± 2.63 a	196 ± 22.5 ab
umamit24-1 UMAMIT24	6.55 ± 1.9 a	23.1 ± 2.02 a	13.4 ± 4.76 a	16.3 ± 3.55 b	20.8 ± 4.36 a	0.61 ± 0.38 a	2.96 ± 0.57 b	5.68 ± 1.19 a	7.99 ± 1.99 a	4.63 ± 1.09 b	1.64 ± 0.86 a	4.14 ± 1.81 a	7.41 ± 0.33 a	0.46 ± 0.26 a	2.96 ± 1.54 a	3.28 ± 1.57 a	6.24 ± 3.38 a	4.77 ± 2.11 a	3.8 ± 1.19 a	137 ± 13.7 c
umamit25-1	6.18 ± 1.89 a	20 ± 5.31 a	18.5 ± 8.86 a	39.1 ± 22.2 ab	32.3 ± 15.4 a	0.99 ± 0.24 a	5.61 ± 2.21 ab	6.77 ± 1.13 a	9.48 ± 2.45 a	8.83 ± 2.05 ab	2.49 ± 1.26 a	6.43 ± 2.98 a	7.2 ± 1.04 a	0.78 ± 0.42 a	4.34 ± 2.15 a	4.76 ± 2.34 a	9.41 ± 4.79 a	5.9 ± 2.39 a	5.78 ± 3.18 a	195 ± 9.67 ab
umamit25-1 UMAMIT25	6.42 ± 1.81 a	20 ± 4.28 a	12.1 ± 5.69 a	33.4 ± 24 ab	22.5 ± 10.7 a	1.11 ± 0.29 a	3.98 ± 0.7 b	5.84 ± 0.98 a	8.76 ± 4.41 a	7.13 ± 2.1 ab	2.22 ± 1.73 a	5.24 ± 3.36 a	8.07 ± 0.98 a	0.74 ± 0.63 a	3.93 ± 3.01 a	4.11 ± 2.87 a	8.19 ± 6.28 a	6.48 ± 4.2 a	4.47 ± 2.41 a	164 ± 19.1 bc

Day 14	ASP	GLU	ASN	SER	GLN	HIS	GLY	THR	ALA	ARG	TYR	VAL	MET	TRP	PHE	ILE	LEU	LYS	PRO	Sum
Wild type	4.11 ± 0.6 ab	10.3 ± 0.66 a	6.03 ± 4.7 a	4.56 ± 0.61 a	7.03 ± 0.92 ab	0.77 ± 0.06 a	2.81 ± 0.13 a	2.78 ± 0.19 ab	7.76 ± 0.65 a	4.54 ± 0.55 a	1.61 ± 0.31 a	3.23 ± 0.59 a	4.58 ± 0.47 a	0.38 ± 0.08 a	2.91 ± 0.69 a	2.66 ± 0.5 a	5.78 ± 1.36 a	3.44 ± 0.59 a	2.44 ± 0.34 a	77.9 ± 5.36 a
umamit24-1	2.98 ± 0.81 b	9.89 ± 1.67 a	1.66 ± 1 a	3.51 ± 0.7 a	3.92 ± 1.22 c	0.53 ± 0.15 b	2.19 ± 0.34 b	1.83 ± 0.21 c	4.8 ± 1.07 b	1.55 ± 0.59 c	0.74 ± 0.38 b	1.53 ± 0.83 b	2.66 ± 0.22 d	0.23 ± 0.12 ab	1.16 ± 0.56 b	1.21 ± 0.67 b	2.02 ± 1.25 b	1.35 ± 0.79 b	0.94 ± 0.31 b	44.8 ± 6.78 c
umamit24-1 UMAMIT24	3.79 ± 0.55 ab	8.86 ± 0.44 a	2.91 ± 1.74 a	3.55 ± 0.88 a	7.81 ± 1.73 ab	0.57 ± 0.05 ab	2.15 ± 0.14 b	2.16 ± 0.54 bc	6.49 ± 2.16 ab	3.74 ± 0.82 a	0.84 ± 0.31 b	1.7 ± 0.64 b	3.31 ± 0.27 cd	0.2 ± 0.06 b	1.39 ± 0.56 b	1.38 ± 0.55 b	2.67 ± 1.19 b	1.87 ± 0.48 b	1.66 ± 0.35 ab	57.1 ± 3.65 b
umamit25-1	3.24 ± 0.1 b	9.91 ± 0.9 a	2.01 ± 0.46 a	4.81 ± 0.21 a	5.69 ± 0.43 bc	0.6 ± 0 ab	2.73 ± 0.22 a	2.8 ± 0.15 ab	5.96 ± 0.34 ab	2.11 ± 0.39 bc	1.27 ± 0.23 ab	2.52 ± 0.51 ab	3.5 ± 0.35 bc	0.31 ± 0.06 ab	2.16 ± 0.52 ab	2.06 ± 0.5 ab	4.21 ± 1.04 ab	2.54 ± 0.5 ab	2.34 ± 0.38 a	60.8 ± 4.58 b
umamit25-1 UMAMIT25	4.63 ± 0.41 a	10.8 ± 1.22 a	2.14 ± 0.19 a	3.48 ± 0.16 a	8.85 ± 1.99 a	0.71 ± 0.12 ab	2.31 ± 0.3 ab	2.94 ± 0.23 a	6.51 ± 0.55 ab	3.54 ± 0.78 ab	1.26 ± 0.2 ab	2.47 ± 0.52 ab	4.34 ± 0.53 ab	0.28 ± 0.05 ab	2.2 ± 0.44 ab	2.12 ± 0.4 ab	4.22 ± 0.85 ab	2.76 ± 0.56 ab	2.44 ± 0.93 a	68.1 ± 5.77 ab

Mature	ASP	GLU	ASN	SER	GLN	HIS	GLY	THR	ALA	ARG	TYR	VAL	MET	TRP	PHE	ILE	LEU	LYS	PRO	Sum
Wild type	1.75 ± 0.85 a	7.02 ± 2.44 a	5.55 ± 0.71 a	3.04 ± 1.5 a	1.18 ± 0.65 a	0.52 ± 0.05 a	2.1 ± 0.63 a	0.58 ± 0.11 a	1.13 ± 0.13 a	4.17 ± 1.95 a	0.17 ± 0.03 a	0.61 ± 0.02 a	0.3 ± 0.03 a	0.14 ± 0.08 a	0.54 ± 0.08 a	0.3 ± 0 a	0.26 ± 0.02 a	0.51 ± 0.14 a	0.79 ± 0.1 a	30.75 ± 3.08 a
umamit24-1	1.91 ± 0.37 a	6.82 ± 1.01 a	5.7 ± 1.22 a	2.47 ± 1.42 a	1.42 ± 0.85 a	0.44 ± 0.09 ab	1.54 ± 0.61 a	0.6 ± 0.13 a	0.98 ± 0.27 a	3.4 ± 1.65 a	0.18 ± 0.02 a	0.59 ± 0.14 a	0.26 ± 0.08 a	0.13 ± 0.04 a	0.51 ± 0.08 a	0.29 ± 0.06 a	0.24 ± 0.06 a	0.48 ± 0.13 a	0.56 ± 0.14 a	28.6 ± 6.1 a
umamit24-1 UMAMIT24	2.31 ± 0.13 a	7.92 ± 0.21 a	3.33 ± 0.1 b	1.15 ± 0.06 a	0.31 ± 0.05 a	0.27 ± 0.01 c	1.11 ± 0.19 a	0.41 ± 0.04 a	0.92 ± 0.07 a	1.39 ± 0.2 a	0.18 ± 0 a	0.5 ± 0.04 a	0.24 ± 0 a	0.16 ± 0.01 a	0.43 ± 0.03 a	0.28 ± 0.03 a	0.19 ± 0.02 a	0.3 ± 0.01 a	0.36 ± 0.37 a	21.85 ± 0.88 a
umamit25-1	1.69 ± 0.17 a	6.87 ± 0.77 a	5.34 ± 0.42 ab	1.57 ± 0.07 a	1.23 ± 0.11 a	0.42 ± 0.07 abc	1.39 ± 0.22 a	0.65 ± 0.01 a	0.75 ± 0.02 a	3.11 ± 0.44 a	0.17 ± 0.01 a	0.61 ± 0.05 a	0.27 ± 0.01 a	0.07 ± 0.01 a	0.44 ± 0.02 a	0.31 ± 0.01 a	0.27 ± 0.01 a	0.47 ± 0.09 a	0.43 ± 0.12 a	26.15 ± 0.94 a
umamit25-1 UMAMIT25	2.03 ± 0.43 a	7.2 ± 0.96 a	4.37 ± 1.16 ab	1.68 ± 0.7 a	0.88 ± 0.49 a	0.34 ± 0.06 bc	1.53 ± 0.54 a	0.49 ± 0.11 a	0.95 ± 0.18 a	2.71 ± 1.37 a	0.19 ± 0.02 a	0.54 ± 0.07 a	0.28 ± 0.04 a	0.13 ± 0.02 a	0.47 ± 0.04 a	0.29 ± 0.07 a	0.24 ± 0.03 a	0.41 ± 0.11 a	0.67 ± 0.51	25.5 ± 4.31 a

Table S5. Amino acid content in 7, 10 and 14 day-old pericarp tissues. Values are expressed in nmol per µg of dry weight. Each sample corresponds to two siliques worth of pericarp tissue coming from the same plant. Means and standard deviations are displayed with n=3. Significant differences ($p<0.05$) are indicated by different letter according to one way ANOVA in conjunction with Tukey's test.

Day 7	ASP	GLU	ASN	SER	GLN	HIS	GLY	THR	ALA	ARG	TYR	VAL	MET	TRP	PHE	ILE	LEU	LYS	PRO	Sum
Wild type	7.19 ± 0.42 a	20 ± 2.23 a	26.5 ± 10 a	117 ± 8.31 a	247 ± 91.9 ab	4.63 ± 0.76 a	20.1 ± 9.67 a	25.8 ± 2.38 a	5.56 ± 0.92 a	66.1 ± 13.6 a	0.55 ± 0.08 a	3.74 ± 0.53 a	4.6 ± 2.29 a	0.5 ± 0.11 a	1.16 ± 0.23 a	3.21 ± 0.47 a	4.81 ± 2 a	4.04 ± 0.86 a	14.8 ± 3.53 a	578 ± 135 a
umamit24-1	9.85 ± 2.57 a	18.7 ± 7.21 a	31.5 ± 10.7 a	77.7 ± 17.3 ab	174 ± 56.2 bc	1.77 ± 0.96 b	3.51 ± 1.82 b	28.2 ± 8.12 a	4.95 ± 0.68 a	47.2 ± 13 ab	0.44 ± 0.13 a	3.21 ± 0.37 a	2.23 ± 0.2 a	0.34 ± 0.17 a	0.64 ± 0.12 ab	2.13 ± 0.52 a	1.74 ± 0.49 b	2.16 ± 0.79 b	19.4 ± 7.64 a	430 ± 94.9 ab
umamit24-1 UMAMIT24	18.8 ± 2.06 b	30.7 ± 4.64 a	25.2 ± 6.1 a	59 ± 15 b	74 ± 22 c	1.75 ± 0.48 b	2.53 ± 2.35 b	36.4 ± 5.27 a	5.94 ± 0.97 a	30.9 ± 13.6 b	0.54 ± 0.2 a	4.03 ± 1.21 a	3.29 ± 1.23 a	0.29 ± 0.18 a	0.5 ± 0.19 b	2.59 ± 0.98 a	1.47 ± 0.39 b	1.78 ± 0.86 b	29.2 ± 8.07 a	329 ± 63.3 b
umamit25-1	10.9 ± 3.51 a	22.8 ± 8.55 a	31.5 ± 12.2 a	87.2 ± 28.6 ab	263 ± 48 ab	3.19 ± 0.59 ab	5.8 ± 5.72 b	25.5 ± 6.06 a	5.95 ± 1.69 a	59.2 ± 17.9 ab	0.57 ± 0.38 a	4.31 ± 1.41 a	1.83 ± 1.08 a	0.33 ± 0.33 a	0.72 ± 0.4 ab	2.86 ± 1.58 a	2.5 ± 1.57 ab	3.08 ± 0.79 ab	14.8 ± 6.46 a	547 ± 92.2 a
umamit25-1 UMAMIT25	11.1 ± 1.75 a	19.4 ± 4.89 a	40.6 ± 12 a	105 ± 19.8 a	306 ± 37.3 a	2.62 ± 1.79 ab	3.32 ± 0.65 b	28.4 ± 2.14 a	4.84 ± 0.85 a	59.4 ± 14.6 ab	0.42 ± 0.05 a	3.51 ± 0.29 a	3.78 ± 0.76 a	0.32 ± 0.1 a	0.64 ± 0.18 ab	2.26 ± 0.29 a	1.74 ± 0.45 b	3 ± 0.13 ab	14.4 ± 6.55 a	612 ± 45.4 a

Day 10	ASP	GLU	ASN	SER	GLN	HIS	GLY	THR	ALA	ARG	TYR	VAL	MET	TRP	PHE	ILE	LEU	LYS	PRO	Sum
Wild type	7.21 ± 0.89 a	24.4 ± 3.49 a	36.9 ± 18.4 a	97.9 ± 13.3 a	242.8 ± 99.6 a	9.67 ± 0.68 a	26 ± 14.7 a	22.5 ± 2.76 a	5.4 ± 0.76 a	161 ± 25.8 a	1.01 ± 0.33 a	5.54 ± 0.88 a	2.71 ± 0.86 a	1.99 ± 1.05 a	2.85 ± 1.6 a	3.93 ± 0.94 a	12.5 ± 8.5 a	20.2 ± 4.16 a	31.5 ± 8.87 a	716 ± 140 a
umamit24-1	12.2 ± 3.08 ab	27 ± 8.26 a	28.1 ± 7.23 ab	57.2 ± 26 ab	134.4 ± 78.3 ab	5.6 ± 1.23 b	6.01 ± 7.77 ab	14.1 ± 3.65 b	6.01 ± 1.74 a	140 ± 34.6 a	0.81 ± 0.32 a	4.35 ± 1.44 a	2.7 ± 2.38 a	1.54 ± 0.87 a	1.86 ± 1.2 a	3.21 ± 1.34 a	7.38 ± 6.34 a	12.1 ± 6.65 a	28.4 ± 5.11 a	494 ± 140 a
umamit24-1 UMAMIT24	20 ± 1.64 b	37 ± 1.36 a	8.6 ± 2.61 b	17.1 ± 2.57 b	14.77 ± 3.17 b	1.94 ± 0.62 c	1.2 ± 0.19 b	10.4 ± 2.38 b	10.6 ± 1.78 b	18.1 ± 7.43 b	0.67 ± 0.28 a	3.61 ± 0.79 a	3.46 ± 1.75 a	1.06 ± 0.79 a	0.7 ± 0.24 a	2.44 ± 1.06 a	1.83 ± 0.6 a	1.8 ± 0.44 b	19.8 ± 4.76 a	175 ± 7.67 b
umamit25-1	15.3 ± 8.93 ab	39 ± 8.91 a	35 ± 11.7 a	55.8 ± 30.6 ab	193 ± 69.2 a	7.57 ± 1.21 ab	9.52 ± 14.5 ab	14.4 ± 2.74 ab	4.16 ± 1.43 a	147 ± 13.2 a	1 ± 0.57 a	4.96 ± 1.36 a	2.66 ± 1.43 a	1.9 ± 0.86 a	2.23 ± 1.16 a	3.52 ± 1.19 a	5.02 ± 4.35 a	14.4 ± 5.41 a	24.6 ± 3.49 a	582 ± 83.9 a
umamit25-1 UMAMIT25	15.6 ± 4.36 ab	31.4 ± 4.2 a	31.3 ± 15.3 ab	49.2 ± 20 ab	176.1 ± 88.3 ab	6.56 ± 2.65 ab	3.8 ± 1.7 ab	17.3 ± 4.91 ab	5.75 ± 1.67 a	129 ± 31.9 a	0.89 ± 0.4 a	5.95 ± 2.22 a	3.14 ± 1.7 a	1.42 ± 0.56 a	1.56 ± 0.5 a	4.15 ± 1.73 a	11.36 ± 3.23 a	12.4 ± 3.52 a	35.5 ± 15.8 a	543 ± 145 a

Day 14	ASP	GLU	ASN	SER	GLN	HIS	GLY	THR	ALA	ARG	TYR	VAL	MET	TRP	PHE	ILE	LEU	LYS	PRO	Sum
Wild type	16.4 ± 1.61 a	34.7 ± 1.17 a	5.76 ± 0.7 a	13.93 ± 1.81 a	12.6 ± 6.13 ab	2.78 ± 0.1 a	3.25 ± 0.15 a	11.7 ± 3.72 a	6.79 ± 0.72 a	29.8 ± 17.2 ab	1.01 ± 0.14 a	3.29 ± 0.29 a	3.33 ± 0.32 a	1.34 ± 0.29 ab	1.51 ± 0.27 a	2.03 ± 0.12 a	3.29 ± 0.09 a	2.04 ± 0.43 a	25.1 ± 5.78 ab	180. ± 36. a
umamit24-1	9.69 ± 2.32 b	27.4 ± 2.86 b	2.13 ± 0.66 c	7.27 ± 1.51 b	4.04 ± 2.85 b	0.75 ± 0.4 b	3.36 ± 0.95 a	4.06 ± 1.2 b	5.64 ± 3.9 a	1.19 ± 1.54 b	0.81 ± 0.5 a	2.06 ± 0.84 b	2.78 ± 2.38 a	0.76 ± 0.45 b	1.3 ± 0.81 a	1.33 ± 0.72 a	2.37 ± 1.24 a	1 ± 1.09 a	4.9 ± 2.28 c	82.9 ± 19.3 b
umamit24-1 UMAMIT24	15.1 ± 1.04 a	32.2 ± 0.97 ab	5.91 ± 1.28 a	12.6 ± 1.59 ab	13.7 ± 1.67 ab	3.43 ± 0.26 a	2.64 ± 0.1 a	11.93 ± 2.38 a	5.31 ± 1 a	44.5 ± 25.5 a	0.89 ± 0.07 a	2.96 ± 0.41 ab	2.41 ± 1.28 a	1.52 ± 0.56 ab	1.48 ± 0.47 a	1.89 ± 0.3 a	2.77 ± 0.58 a	2.33 ± 1.14 a	26.15 ± 4.72 ab	189. ± 22.2 a
umamit25-1	14.2 ± 1.57 a	30.7 ± 4.11 ab	3.08 ± 1.43 bc	10.8 ± 2.4 ab	5.48 ± 1.67 b	1.25 ± 0.54 b	3.32 ± 0.56 a	8.03 ± 2.28 b	5.94 ± 1.41 a	2 ± 2.43 b	0.9 ± 0.25 a	2.54 ± 0.58 ab	3.27 ± 0.37 a	1.35 ± 0.36 ab	1.47 ± 0.7 a	1.66 ± 0.49 a	2.68 ± 0.8 a	1.06 ± 0.6 a	18.41 ± 7.67 b	118. ± 22 b
umamit25-1 UMAMIT25	14.9 ± 1.44 a	29.6 ± 3.73 ab	5.1 ± 1.46 ab	11.1 ± 0.64 a	17.9 ± 9.56 a	2.84 ± 1.12 a	3.05 ± 0.49 a	12.3 ± 1.91 a	5.86 ± 0.63 a	35.2 ± 19.3 ab	0.97 ± 0.01 a	2.82 ± 0.22 ab	3.41 ± 0.06 a	1.72 ± 0.12 ab	1.2 ± 0.38 a	1.75 ± 0.09 a	2.67 ± 0.15 a	1.38 ± 0.28 a	33.3 ± 8.29 a	187 ± 36.9 a
